# Effects of Fabrication Conditions on Structure and Properties of Mechanically Prepared Natural Silk Web and Non-Woven Fabrics

**DOI:** 10.3390/polym13101578

**Published:** 2021-05-14

**Authors:** Yeon-Su Bae, In-Chul Um

**Affiliations:** 1 Department of Biofibers and Biomaterials Science, Kyungpook National University, Daegu 41566, Korea; umic91@naver.com; 2 Institute of Agricultural Science and Technology, Kyungpook National University, Daegu 41566, Korea

**Keywords:** silk non-woven fabric, sericin, reeling, hot press

## Abstract

In this study, natural silk web and natural silk non-woven fabric were prepared mechanically using the binding character of the sericin in silk. The effect of process variables on the preparation, structure, and properties of the silk web and the non-woven fabric was examined. The reeling velocity affected the morphology and mechanical properties of the web but had almost no influence on the crystalline structure of the silk. From the viewpoint of reel-ability and the mechanical properties (work of rupture) of silk web, a reeling velocity of 39.2 m/min represented the optimal processing velocity. The porosity and swelling ratio of the silk web decreased slightly with increasing reeling velocity. Furthermore, the reeling bath temperature had a significant effect on the reel-ability of silk filaments from a silkworm cocoon. Bath temperatures ≥50 °C yielded good reel-ability (>900 m reeling length). The porosity, swelling ratio in water, and mechanical properties of the silk web and silk non-woven fabric changed only slightly with the reeling bath temperature but changed significantly with the hot press treatment. The hot-pressed silk web (i.e., silk non-woven fabric) exhibited higher tensile strength as well as lower elongation at break, porosity, and swelling ratio than the silk web.

## 1. Introduction 

Silk is a naturally occurring biomaterial composed of fibroin and sericin, and it has attracted significant attention, owing to its unique properties including excellent biocompatibility [1,2,3] as well as good cell adhesion and growth [4,5,6] and good biodegradability [7,8]. Silk fabricated in porous forms such as electro-spun silk web and sponge can hold fluid in pores, and cells attach to and grow easily in these pores [9,10,11]. Therefore, porous silk materials have been used to develop (for example) membranes for guided regeneration [12,13,14], nerve conduits [15,16], artificial bones [17,18], wound dressings [19,20], and drug delivery [21]. 

However, the use of regenerated electro-spun silk web and silk sponge in biomedical and cosmetic fields has been quite limited. That is, the fabrication process of the web and sponge consists of many steps (e.g., degumming, drying, dissolution, dialysis, and electro-spinning (or freeze-drying)) and is, hence, both time-consuming and costly [22,23,24,25,26]. Another drawback is that during the degumming and regeneration processes, the molecular weight (MW) of the silk decreases (due to molecular degradation), and the highly crystallized silk structure is disrupted [27,28,29,30,31]. This results in a lack of sufficient mechanical properties of the resultant, regenerated porous materials for a certain application. Moreover, the electro-spinning rate of silk is still relatively low (0.2–3.0 mL/h) and therefore, mass production of electro-spun silk web is difficult, although many studies have focused on improving the spinning rate [22,32,33,34]. 

To overcome these problems, in the previous study [35], a new, natural silk non-woven fabric was prepared using a simple method to exploit the natural binding character of sericin. This method consisted of winding, wetting, and hot press processes to maximize the adhesive character of sericin. The winding process, which was conducted manually in the study, was helpful in determining whether the preparation of a new natural silk non-woven fabric was possible through a simple method (i.e., without regeneration processes). The remarkably improved properties of the non-woven fabric were revealed in the study. That is, all silk non-woven fabrics exhibited a good cell viability of silk regardless of sericin content (8~26%) and excellent tensile strength (max. 50 MPa), owing to its raw silk filaments (with high MW and crystallinity) compared to that of electro-spun silk web (10~11 MPa). 

However, in the previous study, the natural silk non-woven fabric was fabricated using a manual method, which is inadequate for mass production. Although the effects of the hot press temperature and sericin content on the silk non-woven fabric were examined, the effects of other process variables remain unexplored. 

In the present study, two types of porous silk materials were prepared mechanically using an electric winder system to develop the mass production method. That is, with the help of this mechanical system, (1) a silk web was prepared (without a hot press treatment) in addition to (2) a silk non-woven fabric (i.e., hot-pressed silk web). Also, the effects of process variables (reeling velocity, reeling bath temperature, and hot press treatment) on the fabrication, structure, and properties of the silk web and silk non-woven fabric were examined to find ways to control the performance of these porous silk materials diversely. It is expected that the mechanically produced silk web and silk non-woven fabric will make mass production of these materials possible, reducing production costs and improving the quality consistency of these materials. Finally, this will lead to the successful application of silk web and silk non-woven fabric to various fields, including cosmetic and biomedical fields.

## 2. Experimental Section

### 2.1. Materials

Baekokjam *Bombyx mori* silk cocoons were kindly provided by Gyeongsangbuk-do Silkworm & Insect Management Center (Sangju, South Korea). The cocoons were dried for 4 h at a high temperature (90 °C) to kill pupa. Subsequently, dried silk cocoons with dead pupa were used for fabrication of a silk web and a silk non-woven fabric.

### 2.2. Fabrication

**Figure 1:**
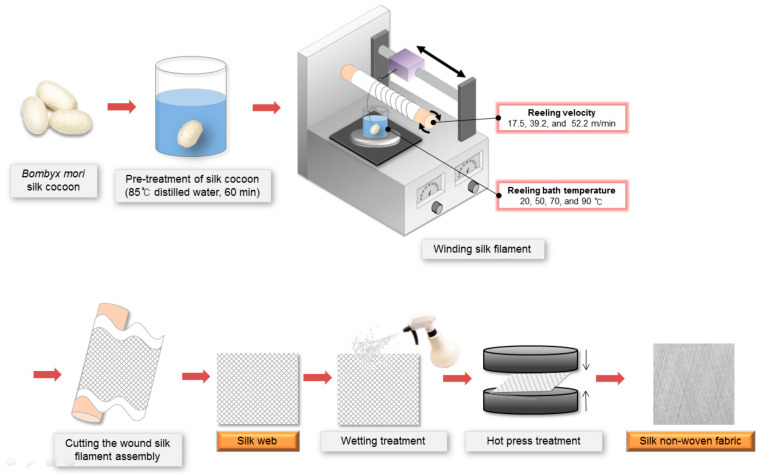
Schematic showing the preparation of the silk web and silk non-woven fabric using an electric winder system.

The fabrication procedure and device used for obtaining the silk web and silk non-woven fabric are presented in Figure 1. During the preparation process, a silkworm cocoon was immersed for 60 min in a bath of distilled water at 85 °C as a pretreatment for swelling the sericin in the cocoon. The cocoon was then moved to a reeling bath at various temperatures (20 °C, 50 °C, 70 °C, and 90 °C) of the silk web manufacturing machine (SWMM-1, Donga Machinery, Namyangju, South Korea). The machine, which was designed in-house, consisted of: (1) an electric winder capable of winding and transverse move functions and (2) a reeling bath. Winding (reeling) and transverse speeds could be varied by the electronic controller in the machine. The external diameter of the roll collector was 43 mm. 

In the present study, a silk web with a cross-over angle of 30° between the constituent silk filaments, as indicated in the left cell of Table 1, was produced. The web was produced by reeling silk filaments into the roll collector with reeling velocities of 17.5 m/min, 39.2 m/min, and 52.2 m/min as well as transverse speeds of 4.7 m/min, 10.5 m/min, and 14.0 m/min. Reeling of silk filament in a cocoon started at the right side of the roll collector (starting point). When the silk filament of a cocoon was consumed by reeling, the next silk cocoon was reeled again from the starting point. After the reeling (winding) of all filaments was completed, the web was obtained by cutting the assembly of filaments wound on the roll collector. 

To fabricate the silk non-woven fabric, the silk web was humidified by spraying with distilled water for 5 min and pressing twice with a hot presser (HK 2008-1-5, Hankuk Industry Co., Gwangju, Korea) at 200 °C for 10 s. A press temperature of 200 °C was used because it was reported to be the optimum condition for the maximum bonding effect of sericin [35]. The silk web became the silk non-woven fabric when the silk filaments in the silk web became strongly bound together via the hot press treatment. To prevent the silk web from adhering to the hot press plates, polyester non-woven fabrics were placed on the top and bottom of the silk web. 

### 2.3. Measurement and Characterization

The external features of the silk web and silk non-woven fabric were photographed using a digital camera (PC1310, Canon, Beijing, China). Moreover, the corresponding morphologies were examined by means of field emission scanning electron microscopy (FE-SEM; S-4800, Hitachi, Tokyo, Japan). The samples were coated with Pt-Pd prior to the FE-SEM observations.

The crystalline structure and crystallinity of the silk web and silk non-woven fabric were examined using the wide-angle X-ray scattering (WAXS) method [27,36,37]. X-ray fiber diffraction patterns were obtained with a VANTEC500 (D8 Discover, Bruker, Karlsruhe, Germany) using CuKα radiation. Similarly, X-ray diffractograms were obtained through 2θ scanning of the X-ray fiber diffraction pattern (irradiation conditions: 50 kV, 1000 μA, and 600 s). 

The reeling length was measured to evaluate the reel-ability of the silk filament from the silkworm cocoon. It was the length of reeled silk filament until the occurrence of the first breakage. The reeling length was calculated from the reeling velocity and reeling time associated with this breakage. 

A universal testing machine (OTT-003, Oriental TM, Ansan, Korea) was used to evaluate the mechanical properties of the silk web and silk non-woven fabric [35,38,39,40,41]. In the case of these silk samples, the test was performed with a 200 kgf load cell at an extension rate of 10 mm/min and a gauge length of 30 mm. The silk samples were cut into 20 mm × 45 mm pieces, and each sample was preconditioned under the standard conditions (i.e., 20 °C and 65% relative humidity) for more than 24 h. In addition, the mechanical test of samples was performed under the standard conditions. The samples were extended in the direction of the center of the acute angle between silk filaments, as expressed as a dotted arrow in the left cell of Table 1. Seven specimens were tested for each condition, and the average and standard deviation were obtained from the five results remaining after the maximum and minimum values were removed. 

To measure the porosity of the silk web and non-woven fabric, the web and fabric were immersed in ethanol of volume (V_1_) for 5 min. After the sample was completely soaked and the ethanol penetrated the sample, the total volume (V_2_) of the ethanol and the sample was measured. The silk sample was removed from the ethanol, and the volume of the residual ethanol volume (V_3_) was recorded. Afterward, the porosity of the sample was determined from Equation (1) [39,40,42]. The average values of the three measurements for each condition were taken for calculation of the porosity:

(1) $$\mathrm{Porosity}\;\left(\%\right)=\frac{V_{1}-{\;V}_{3}}{V_{2}-{\;V}_{3}}\times 100$$

To obtain the swelling ratio in water, the silk web and non-woven fabric were soaked in distilled water for 4 h at room temperature. After the silk sample was fully soaked, the excess water on the surface of the sample was removed within 1 min. The weight of the swollen silk sample (W_s_) was measured, and then the weight of the dried silk sample (W_d_) was measured using a moisture analyzer (XM60, Precisa, Dietikon, Swiss). The swelling ratio was determined from Equation (2) [39,40]. The swelling measurement was performed three times, and the average swelling ratio was calculated by averaging the values obtained from these three measurements:

(2) $$\mathrm{Swelling}\;\mathrm{ratio}\;\left(\%\right)=\frac{W_{s}-{\;W}_{d}}{W_{d}}\times 100$$

## 3. Results and Discussion

### 3.1. Effect of Reeling Velocity

Unlike the previous study [35], in the present study, the silk web was mechanically prepared using an electric winder system wherein various reeling velocity values could be employed. The web was fabricated at different reeling velocities. As shown in Table 1, the web could be prepared without a hot press treatment, owing to the binding effect of swollen sericin. That is, the sericin in silk becomes swollen during pretreatment at 85 °C and in reeling baths at 20~90 °C. The swollen sericin of a silk filament binds easily with the swollen sericin in neighboring silk filaments, allowing the formation of a silk web without hot pressing of the filaments. 

Regardless of the reeling velocity, extremely similar features and luster were observed for all the silk webs (Table 1). However, the webs were characterized by differing morphologies, as revealed by means of FE-SEM (Table 2). That is, as the reeling velocity was increased, the surface roughness of the silk filament in the silk web increased, and a number of marks (shown as white dashed circles) appeared (see Table 2). These marks may have resulted from the detachment of each filament from neighboring filaments. That is, when the silk filament was reeled from the silk cocoon, the filament became detached from neighboring filaments because the sericin of the filaments became swollen in the reeling bath at elevated temperatures. The silk filament was more rapidly detached at higher reeling velocity (than at lower reeling velocity), leading to an increase in the number of detachment-induced marks. 

**Figure 2:**
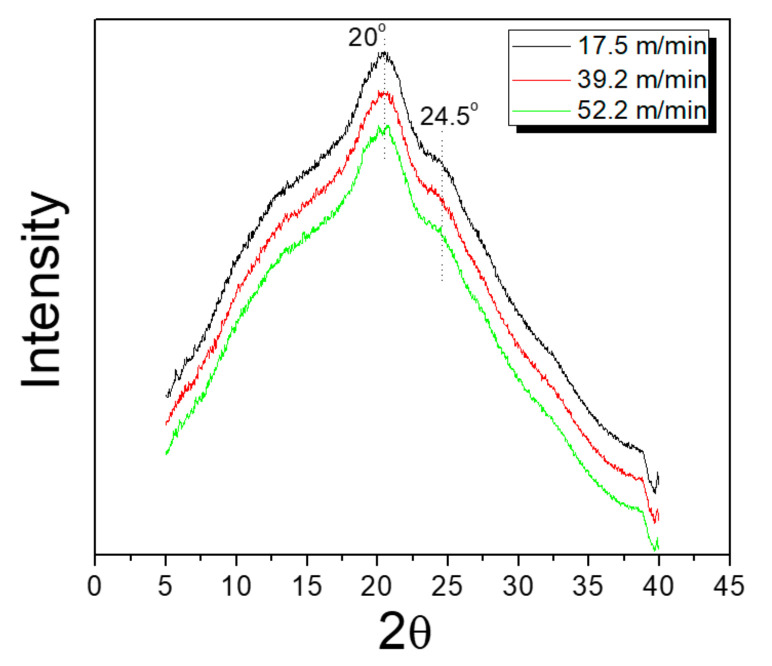
X-ray diffractograms of silk webs prepared at various reeling velocities.

XRD measurements were performed on the silk webs and non-woven fabrics to determine their crystalline structure and crystallinity because these characteristics affected the physical properties of the silk webs and non-woven fabrics. The reeling tension increased with increasing reeling velocity and therefore, the reeling velocity may have influenced the crystallinity of a silk web. Therefore, XRD measurements were performed on webs obtained at different reeling velocities (see Figure 2). 

Regardless of the reeling velocity, a crystalline peak at 20° and a shoulder at 24.5° (attributed to the β-sheet crystallites of silk protein) occurred in the XRD diffractogram of each silk web [27,36,43,44,45]. Furthermore, the XRD peak intensity remained almost constant with changing reeling velocity. Natural silk filaments in the silkworm cocoon consisted of β-sheet crystallites and hence, the occurrence of such crystallites in the silk webs was unsurprising. This result indicated that the reeling tension used in this study had no effect on the crystalline structure (i.e., β-sheet crystallites) and crystallinity of the silk webs. 

**Figure 3:**
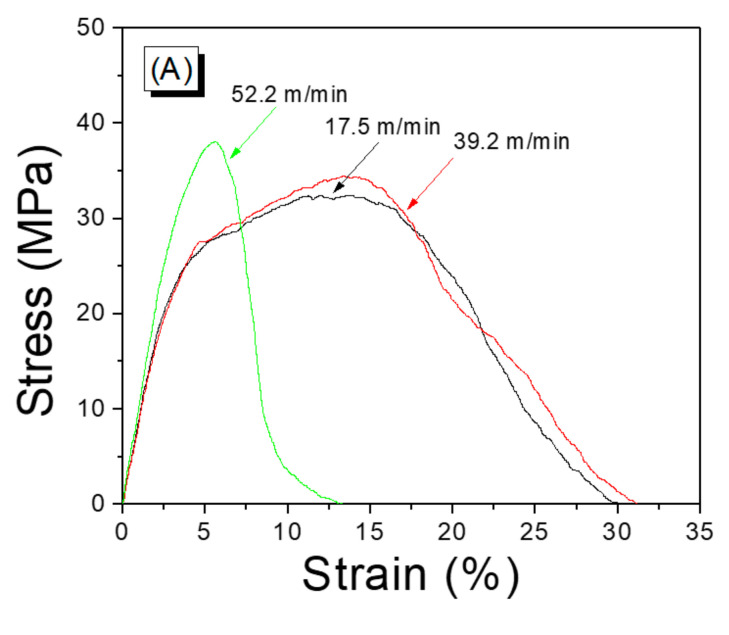

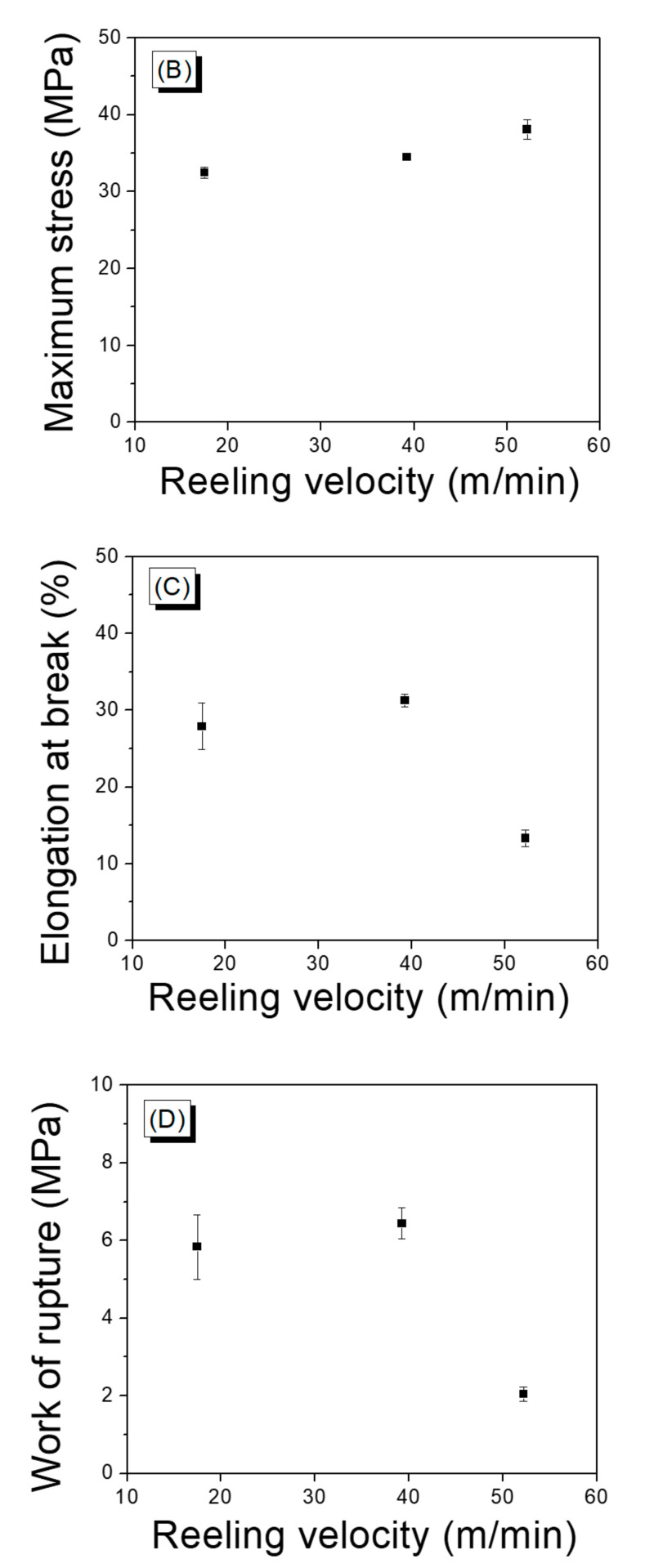
(**A**) Representative stress–strain curve, (**B**) tensile strength, (**C**) elongation at break, and (**D**) work of rupture determined for silk web prepared at various reeling velocities. Reeling bath temperature was 50 °C.

The mechanical properties of a silk web are vital for cosmetic and biomedical applications. Therefore, in this work, these properties are measured as a function of the reeling velocity (see Figure 3). As shown in the stress–train (S–S) curve (Figure 3A), the web obtained at a reeling velocity of 17.5 m/min exhibited ductile behavior characterized by a high elongation at break (30%). The S–S curve of the web remained almost unchanged until a velocity of 39.2 m/min was reached. However, a further increase in the reeling velocity (52.2 m/min) resulted in a behavior of brittle material characterized by a significant decrease in the elongation at break (13%). The maximum stress of the silk web increased slightly with increasing reeling velocity (Figure 3B). The elongation at break and work of rupture changed only modestly up to velocities of 39.2 m/min and decreased significantly at 52.2 m/min (Figure 3C,D). 

The changes in the mechanical properties were related to different macro-structures of the silk web induced by the different reeling velocities. That is, as the reeling velocity was increased, the silk filaments were wound more tightly (than at lower velocities) on the electric winder because the tension of the filaments increased with increasing reeling velocity. A tighter winding of the filaments resulted in a more compact structure of the web (than a looser winding), leading to an increase in the maximum stress and a decrease in the elongation at break. This implies that, depending on the application, we can manipulate the mechanical properties of a silk web by controlling the reeling velocity. 

The production rate of the silk web improved with increasing reeling velocity and the work of rupture as well as the elongation of the web decreased considerably at 52.2 m/min. This suggested that 39.2 m/min was the optimal reeling velocity for silk web production. Therefore, a velocity of 39.2 m/min was used in the subsequent examinations. 

A porous fibrous material becomes more important because of its holding ability of various things including fluids and cells. In addition, some researchers have conducted theoretical works to understand the properties of porous fibrous materials [46,47]. 

**Figure 4:**
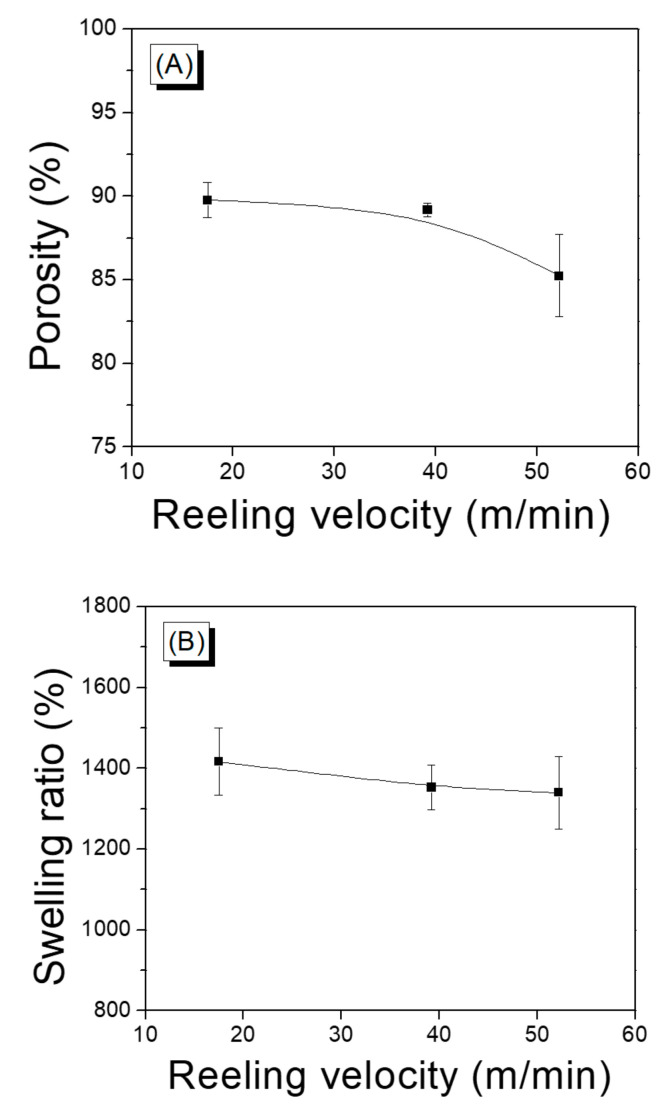
(**A**) Porosity and (**B**) swelling ratio of silk web prepared at various reeling velocities. Reeling bath temperature was 50 °C.

The porosity of an electro-spun web is important because it influences the cell viability [48], and the porosity of an electro-spun silk web determines the mechanical properties of the silk [31,49]. Based on the significance of porosity, the effect of reeling velocity on the porosity of the silk web was evaluated, and the result is shown in Figure 4A. The porosity decreased from 89.8% to 85.2% with increasing reeling velocity. This might be due to the different compactness of silk web that resulted from different reeling velocities. That is, when the reeling velocity was increased, the silk filaments were wound more tightly (than at lower velocities), resulting in a more compact structure of the web, as previously mentioned. The increase of compactness led to the decrease of pores in the silk web. The significant decrease in porosity at 52.2 m/min was quite similar to the trends describing the elongation and work of rupture obtained for the web at 52.2 m/min. This confirmed that different mechanical properties of the silk web were correlated with the compactness (i.e., porosity) of the web.

The swelling ratio of the silk web in water decreased slightly from 1416% to 1340% by increasing the reeling velocity (Figure 4B). This was attributed to the change in porosity (or compactness) of silk web with reeling velocity. That is, as the porosity was reduced by increasing the reeling velocity, the space (pore) capable of holding water was reduced, leading to a decrease in the swelling ratio in water. It was noteworthy that the silk webs were capable of holding water amounts that were considerably greater (13-fold) than the silk weight.

In a previous study [35], the silk non-woven fabric was prepared manually and therefore, production of the fabric was time consuming. Breakage of the silk filament was prevented during the manual reeling process because the reeling was conducted slowly and carefully. However, in the present study, the silk web was mechanically fabricated using an electric winder system wherein the reeling velocity employed (17.5 m/min~52.2 m/min) was much higher than that of the manual method (<5 m/min). The production process of the silk web stopped when the reeling of the filament was interrupted due to the breakage of the filament. That is, the reel-ability, which can be expressed as the reeling length, played a key role in the productivity of silk web via this mechanical production system. 

**Figure 5:**
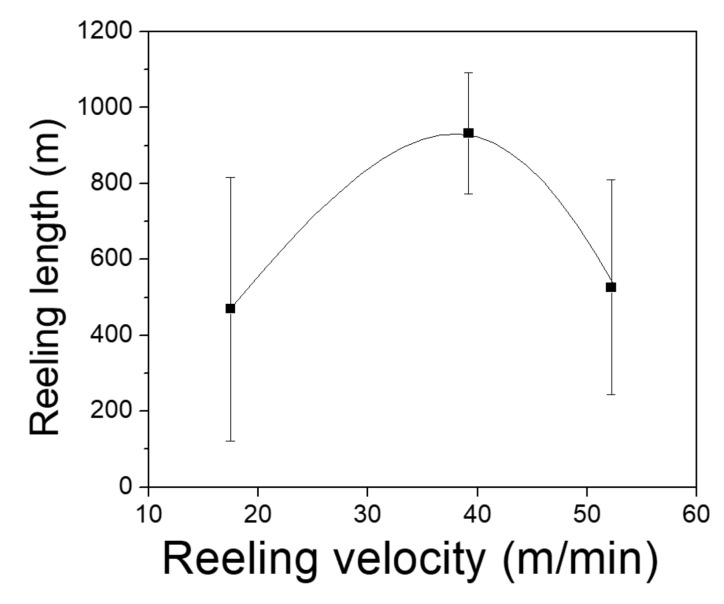
Effect of reeling velocity on the reel-ability of silk filament from silk cocoons (*n* = 20). Reeling bath temperature was 50 °C.

Figure 5 shows the reeling length of the silk filament as a function of the reeling velocity. As the figure shows, the mean reeling length (469 m) of the filament at a reeling velocity of 17.5 m/min increased to 933 m at 39.2 m/min and then decreased to 527 m at 52.2 m/min. This indicated that the reel-ability of the silk filament from the cocoon in the electric winder system was associated with an optimum reeling velocity (39.2 m/min in this study). The mean length of silk filaments that could be extracted from the silk cocoons used in this study was 1007 m. Therefore, the mean reeling length (933 m) at 39.2 m/min indicated that 93% of the silk filaments could be reeled and extracted from the silk cocoon until the first breakage occurred. That is, the silk filament in the cocoon could be efficiently utilized in the fabrication of a silk web by using the optimum reeling velocity (39.2 m/min). 

### 3.2. Effect of Reeling Bath Temperature and Hot Press Treatment

In this study, the silk web was prepared using the binding character of sericin [35]. To use the binding effect of sericin, the sericin should be properly swollen. Therefore, the control of the swelling condition for the sericin played an important role in the productivity of a silk web and the properties of the resultant web. The sericin in the silk filament was swollen in the reeling bath and hence, various reeling bath temperatures were used to fabricate the web. Subsequently, the effects of the bath temperature on the structure and properties of the web were evaluated. The final product, silk non-woven fabric, was prepared by pressing the silk web at the elevated temperature (i.e., 200 °C). The sericin was deformed and bound the filaments strongly together through the hot press treatment. Finally, a silk web (untreated silk web) became a silk non-woven fabric (hot-pressed silk web). The hot press would affect the structure and properties of these porous silk materials (web and non-woven fabric) [35]. Therefore, we compared the structure and properties of the silk non-woven fabric with those of the silk web to examine the effect of the hot press treatment on the silk porous materials. 

**Figure 6:**
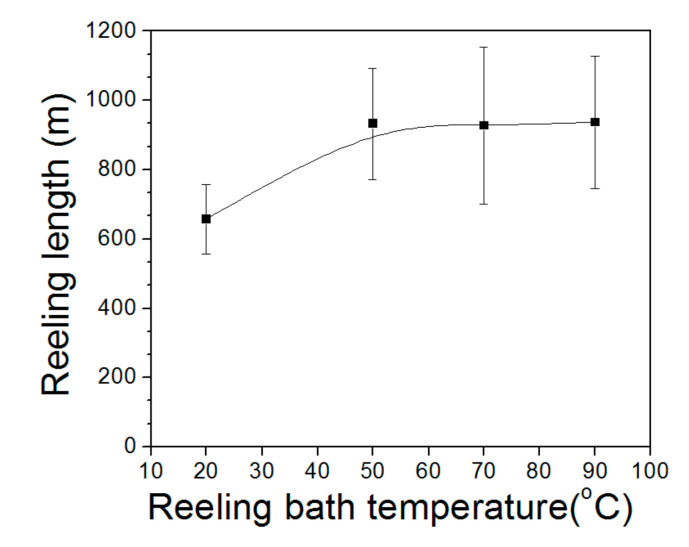
Effect of reeling bath temperature on the reel-ability of silk filament from silk cocoons (*n* = 20). The reeling velocity was 39.2 m/min.

The reel-ability determined the productivity of the silk web and silk non-woven fabric. Therefore, the reeling length was measured to determine the effect of reeling bath temperature on the reel-ability of the silk cocoon for fabrication of the web, and the results are shown in Figure 6. The mean reeling length at a reeling bath temperature of 20 °C was 657 m. At a bath temperature of 50 °C, the reeling length increased to 933 m and remained approximately constant (927 m–936 m) thereafter. This indicated that the reeling of the silk filament from the cocoon was favored at temperatures above 20 °C until a critical temperature (i.e., 50 °C in this condition) was reached. This result might be attributed to (1) the reel-ability was related to the swelling of the sericin, and (2) the swelling of the sericin changed depending upon the bath temperature. That is, as bath temperature was increased, the degree of swelling of the sericin was increased. With increased swelling of the sericin, the filament became more easily detached from neighboring filaments (than at lower levels of swelling), resulting in less breakage of the filaments during the reeling. 

It has been reported that reeling of the silk filament is strongly affected by temperature [50,51]. In this study, the reel-ability of the silk increased with increasing temperatures of up to 50 °C, implying that optimal swelling of the sericin occurred at 50 °C or above. Negligible increase in the reel-ability at temperatures above 50 °C indicated that the sericin was properly swollen at 50 °C or above, thereby leading to maximum reel-ability. Furthermore, reeling lengths of ~930 m indicated that 93% of the extractable silk filament of the silkworm cocoon was reeled at 50 °C or above. Therefore, the optimal swelling of sericin and consequently, the optimal reel-ability, were realized at 50 °C or above. 

Table 3 shows pictures of the silk web and silk non-woven fabric prepared at various reeling bath temperatures. The non-woven fabrics obtained at different temperatures were barely distinguishable. However, the hot press treatment affected the appearance of these porous silk materials. That is, the non-woven fabric seemed to be more compact than the web, which appeared to be looser and rougher than the non-woven fabric. Moreover, the good luster exhibited by the webs was lacking in the case of the non-woven fabrics. 

The effects of the reeling bath temperature and hot press treatment on the morphologies of the silk webs and silk non-woven fabrics were investigated in further detail via FE-SEM (see Table 4). The morphologies of the webs and non-woven fabrics remained almost unchanged with the bath temperature, indicating that the temperature had no effect on the morphology. However, the hot press treatment led to significant changes in the morphology of the web. Regardless of the reeling bath temperature, each silk web consisted of straight and regularly arranged silk filaments, whereas the non-woven fabric consisted of bent and twisted filaments. This resulted from the hot press treatment process. As mentioned in the experimental section, the silk non-woven fabric was prepared by wetting the silk web with spraying water and then hot pressing the web to maximize the binding strength of the sericin. During the treatment, water evaporated quickly from the silk filaments, leading to contraction and bending of the filaments. In addition, the good luster of the web (Table 3) was attributed to the straight and regularly arranged filaments (Table 4), considering that luster resulted from the regular reflection of light against the filaments. Furthermore, the lack of luster of the fabric was attributed to irregular (i.e., bent and twisted) arrangement of the filaments. 

**Figure 7:**
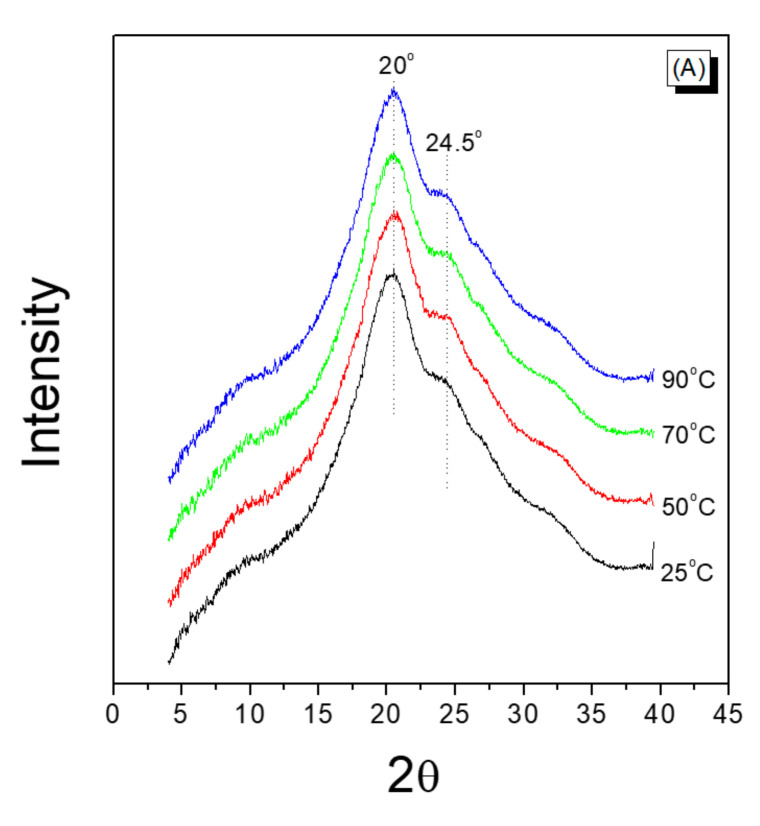

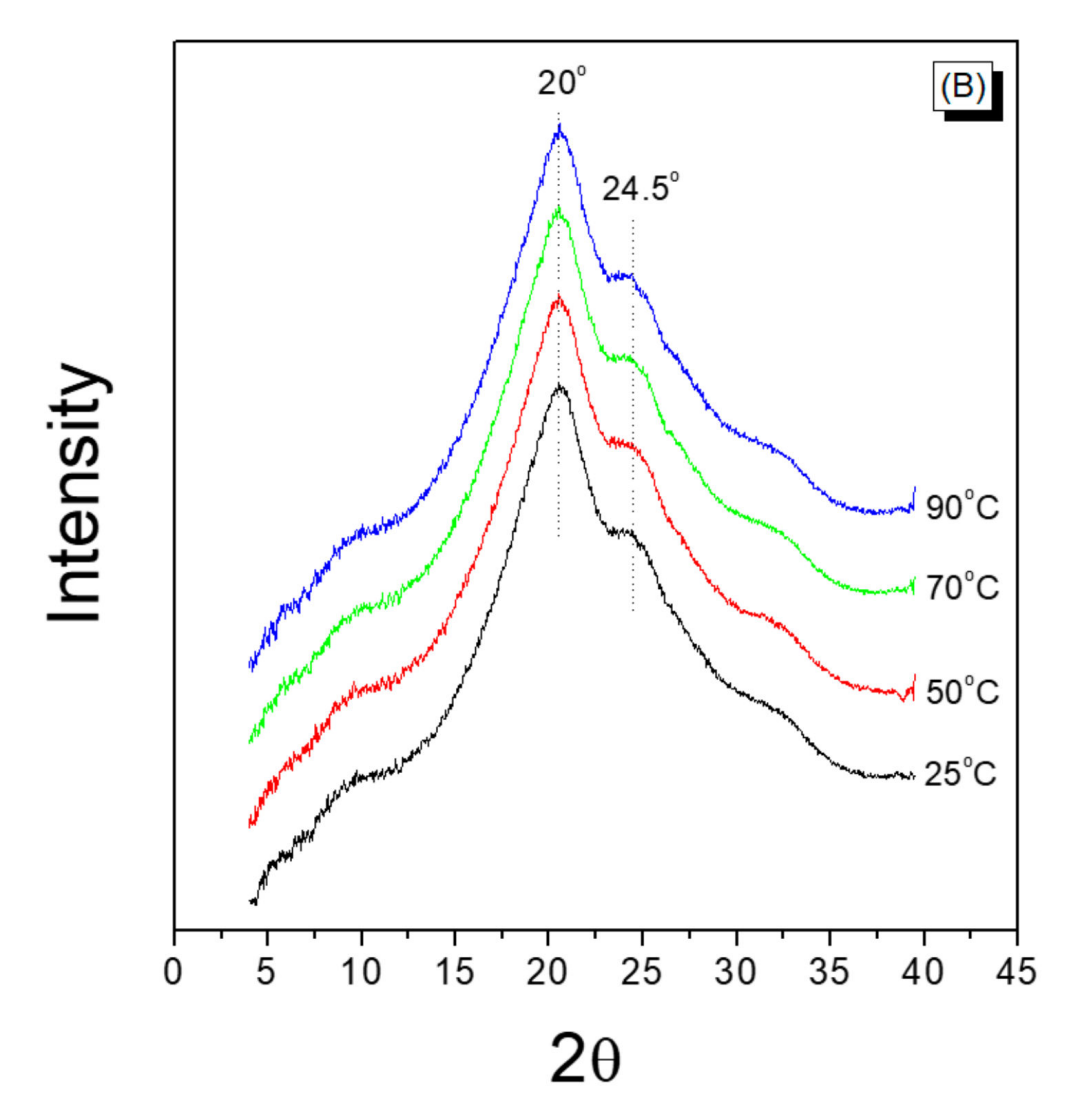
X-ray diffractograms of (**A**) silk web and (**B**) silk non-woven fabrics prepared at various reeling bath temperatures. The reeling velocity was 39.2 m/min.

Figure 7A shows the XRD results of the silk web obtained at different reeling bath temperatures. A crystalline peak at 20° and a shoulder at 24.5° (attributable to β-sheet crystallites) occurred in each case [27,36,43,44,45]. As the bath temperature was increased to 50 °C, the shoulder at 24.5° became slightly more pronounced than at lower temperatures and remained unchanged thereafter. Kim et al. reported that the shoulder at 24.5° in degummed silk became more evident with increasing amounts of degumming agent and the degumming ratio. In addition, the crystallinity index calculated from FTIR spectra reconfirmed the increase of crystallinity of degummed silk by increasing the degummed ratio [27]. Therefore, it can be said that this more evident shoulder at 24.5° in Figure 7A is indicative of an increase in β-sheet crystallite formation. 

For the silk non-woven fabric (Figure 7B), the shoulder occurring at 24.5° was more pronounced than that of the silk webs, regardless of the reeling bath temperature. This indicated that additional β-sheet crystallites of silk were formed, owing to the hot press treatment. The X-ray diffractogram of the fabric exhibits no dependence on the bath temperature. 

It was interesting to note that (1) additional crystallization was induced by the hot press treatment; (2) more β-sheet crystallites were formed in the silk web at a reeling bath temperature of 50 °C or above; and (3) the reeling bath temperature had no effect on the crystallinity of the silk non-woven fabric. 

First, the β-sheet crystallization of silk induced by the hot press treatment was related to the crystallization character of the silk. The silk was composed of silk fibroin (SF) and sericin, and when treated at relatively high temperatures (e.g., 200 °C), the SF underwent crystallization [52,53]. However, the β-sheet crystallite formation of sericin was disrupted at high temperatures [35,38]. That is, opposing crystallization trends were observed for SF and sericin in silk subjected to hot pressing at high temperatures (i.e., 200 °C). A previous study [35] utilizing FTIR-ATR measurements reported that the β-sheet crystallite formation of sericin was disrupted by increasing the press temperature. This conclusion was based on the fact that the ATR technique provided quite sensitive examination of the sample surface, and sericin was present in the surface of the silk filament. However, XRD measurements performed in the present study revealed a different trend: β-sheet crystallite formation occurred preferentially at a press temperature of 200 °C. The XRD measurement reflected the crystalline structure of the entire sample (not only the surface) because the X-ray passed through the entire measurement region of the sample. Furthermore, silk contains more amount of fibroin (70–80%) than sericin (20–30%) [27,38,45,54]. Changes in the crystallinity of the silk (induced by the hot press) were therefore more attributable to the changes in SF rather than to the changes in sericin. That is, the increase in the crystallization of the structure composing the silk resulted from the increase in the crystallinity of SF during hot pressing at 200 °C. 

Second, the increase in the formation of β-sheet crystallites in the silk web at 50 °C or higher temperatures resulted possibly from the crystallization of the sericin via swelling and dissolution. That is, β-sheet crystallization of sericin occurs when swollen or dissolved sericin in water is dried [37,39,55]. Sericin exists in the amorphous state in raw silk but becomes β-sheet crystallized after swelling or dissolution and subsequent drying. The sericin of the silk filaments at a reeling temperature of 25 °C was less swollen than that at higher reeling temperatures (i.e., 50 °C–90 °C), resulting in less β-sheet crystallization. This result was consistent with that obtained for the reel-ability of the filaments (Figure 6). That is, less swollen sericin at a reeling bath temperature of 25 °C led to lower reel-ability and less β-sheet crystallization than at other temperatures (i.e., 50 °C–90 °C). No crystallization of the SF was observed with treatment in water at temperatures below 95 °C. Therefore, increased crystallization of the silk web at relatively high reeling bath temperatures (50 °C–90 °C) was attributed to the drying of more swollen sericin at higher temperatures (than at lower temperatures). 

Last, the reeling bath temperature had no effect on the crystallinity of the silk non-woven fabric. As mentioned above, the different amounts of crystalline regions of the sericin were formed depending on the reeling bath temperature, while the crystalline regions of the SF did not change depending on the reeling bath temperature. However, when the silk web was hot pressed to be non-woven fabric, the differently crystallized sericin was all disrupted with the heat (i.e., 200 °C), and the SF became more crystallized with the heat. This resulted in the same level of crystallinity of silk non-woven fabric regardless of reeling bath temperature. 

Table 5 shows the X-ray fiber diffraction patterns of the silk web and silk non-woven fabric prepared at various reeling bath temperatures. Regardless of the temperature, the pattern obtained for each web consisted of four strong X-ray diffraction spots at 2θ = 20° and 24.5°, which were attributed to β-sheet crystallites. These spots resulted from the straightness of the silk filaments, which were arranged with a crossing angle of 30° in the web, as shown by the FE-SEM results in Table 4. The pattern corresponding to the non-woven fabrics consisted of two XRD arcs (i.e., the diffraction spot became broadened to a diffraction arc) at 2θ = 20° and 24.5°. This resulted from the fact that the silk filaments in the fabric became bent and twisted during the hot press treatment (Table 4), as previously mentioned. That is, as the silk filament became bent and twisted, the azimuthal angle of diffraction became more diverse, leading to the change in the diffraction pattern from the spot to the arc. 

**Figure 8:**
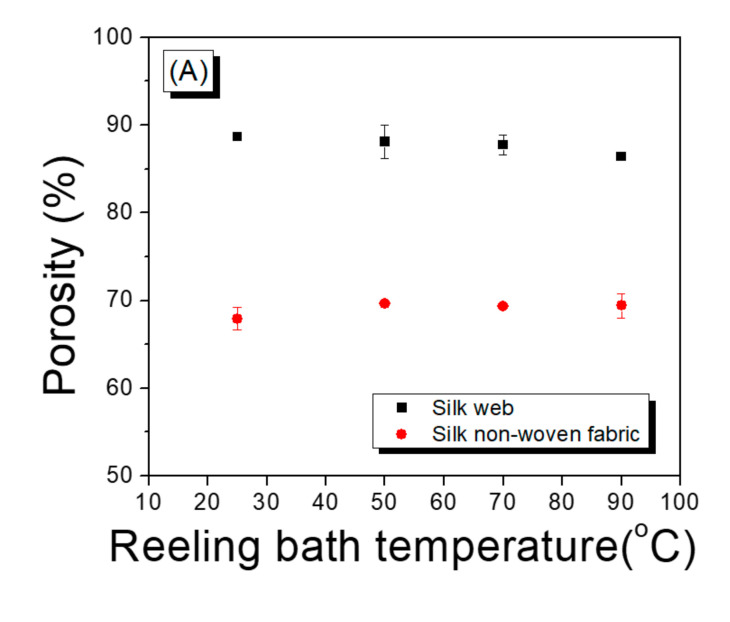

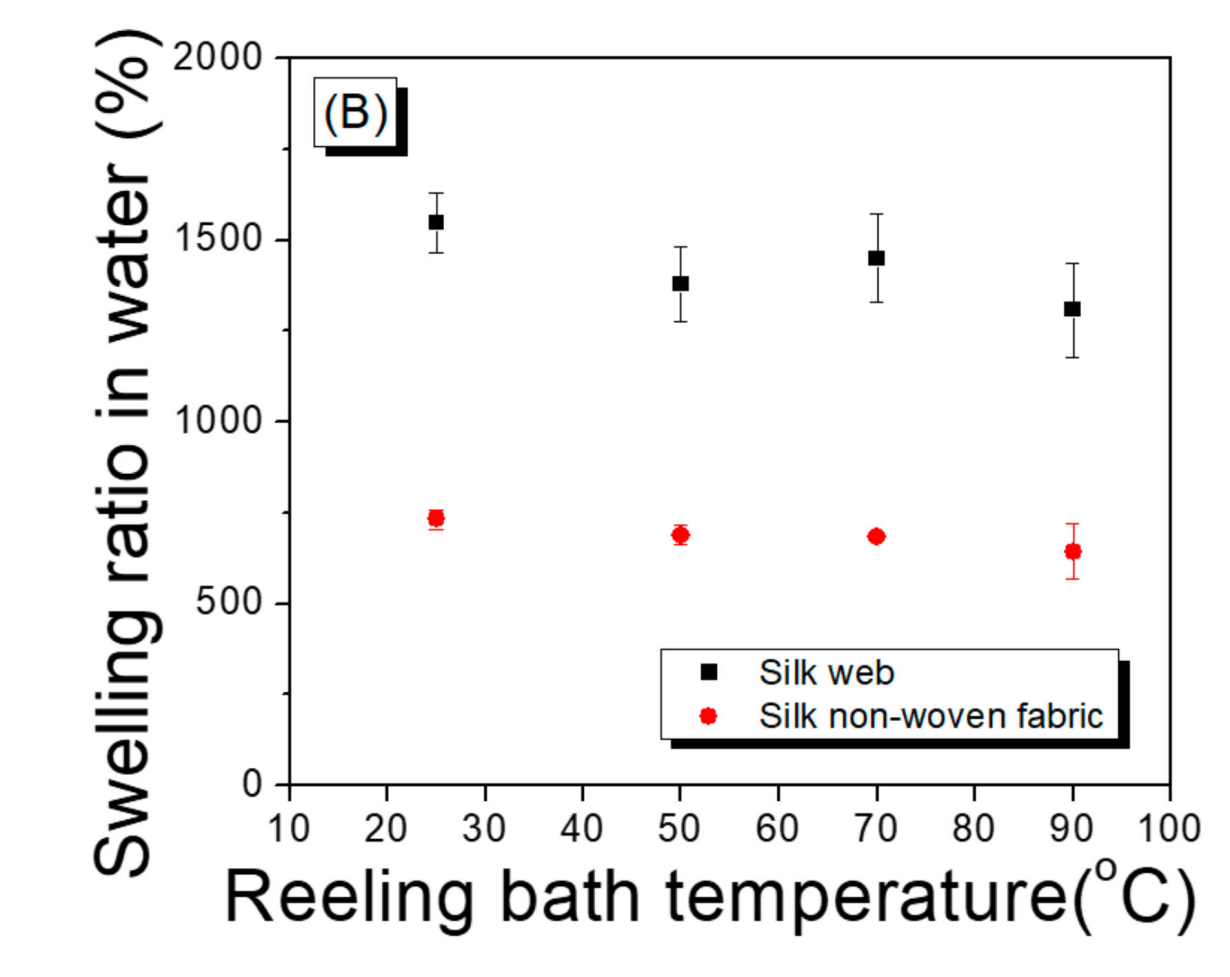
(**A**) Porosity and (**B**) swelling ratio of silk web and silk non-woven fabrics prepared at various reeling bath temperatures. The reeling velocity was 39.2 m/min.

Figure 8 shows the porosity and swelling ratio (in water) of the silk web and silk non-woven fabrics. Regardless of the reeling bath temperature, the silk non-woven fabrics were characterized by lower porosities and swelling ratios than the silk webs, owing to the compression effect induced by the hot press treatment. That is, when the silk web underwent hot pressing, the compactness of the morphological structure increased and the space (pore) between the silk filaments decreased, leading to a reduction in the porosity. The porous material could hold water in the void space (i.e., pores). Therefore, the lower swelling ratio of the silk non-woven fabric (compared with that of the web) was attributed to a decrease in the porosity of the non-woven fabric, owing to the hot press treatment. This result was quite consistent with the good correlation between the porosity and swelling ratio of a silk porous material considered in a previous study [40]. 

In the case of the silk web, the porosity and swelling ratio decreased slightly with increasing reeling bath temperature. This may have resulted from the difference in the swelling of the sericin at different reeling bath temperatures. That is, as the temperature was increased, the swelling of the sericin contained in the silk (in the reeling bath) increased, and the swollen state lasted longer (than the non-swollen state) during the winding process. Silk filaments with more swollen sericin were more flexible and deformable on the winder (than those without swollen sericin), resulting in tighter stacking of the filaments on the winder. This led to a slight decrease in the porosity of the silk web. 

For the silk non-woven fabrics, the porosity and swelling ratio in water almost did not change with reeling bath temperature, indicating the reeling bath temperature did not affect the porosity and the swelling ratio of silk non-woven fabric. This result was consistent with that of the FE-SEM in Table 4. As previously mentioned, the void space between silk filaments in the silk web was minimized by the hot press treatment. The porosity level of the web varied with the reeling bath temperature. However, the different porosity levels of the web were reduced to the minimum level by the hot press, resulting in the same porosity level in the non-woven fabrics regardless of reeling bath temperature. Also, owing to the positive relationship between the porosity and the swelling ratio, the swelling ratio of the silk non-woven fabric remained unchanged, despite variations in the reeling bath temperature. 

**Figure 9:**
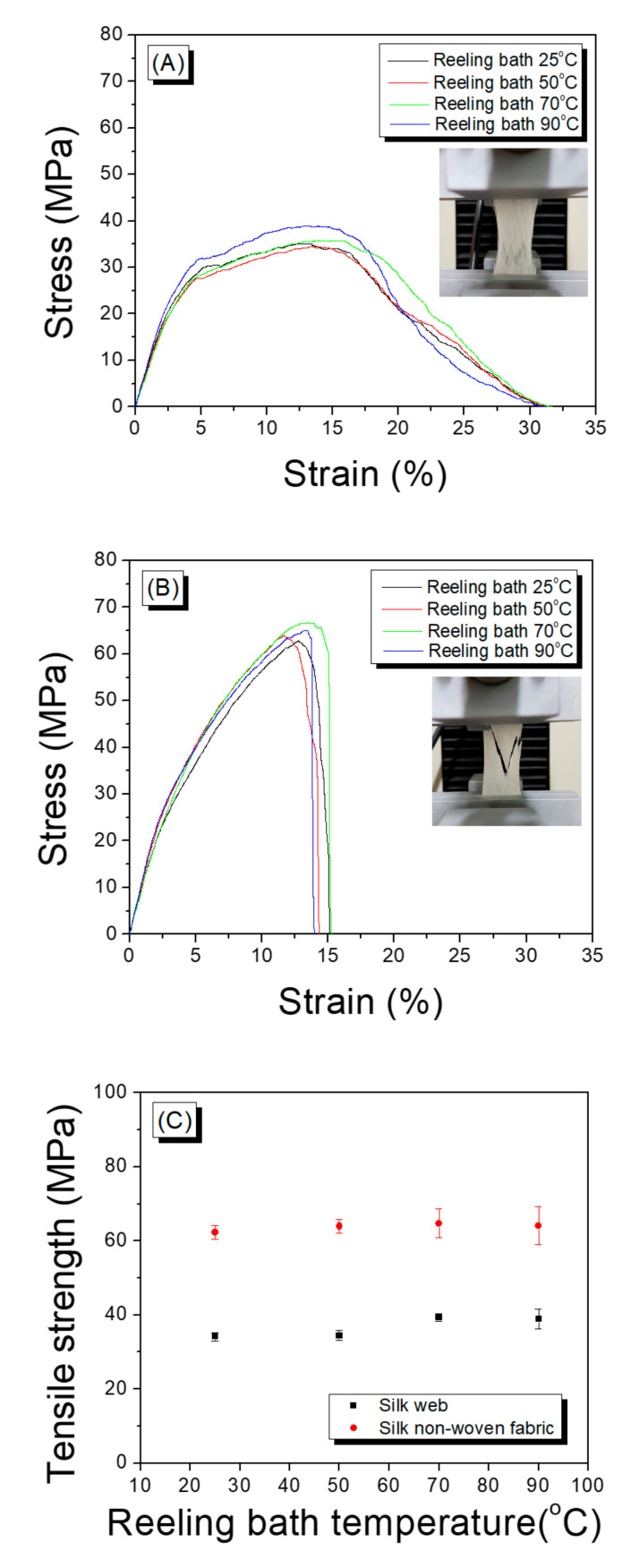

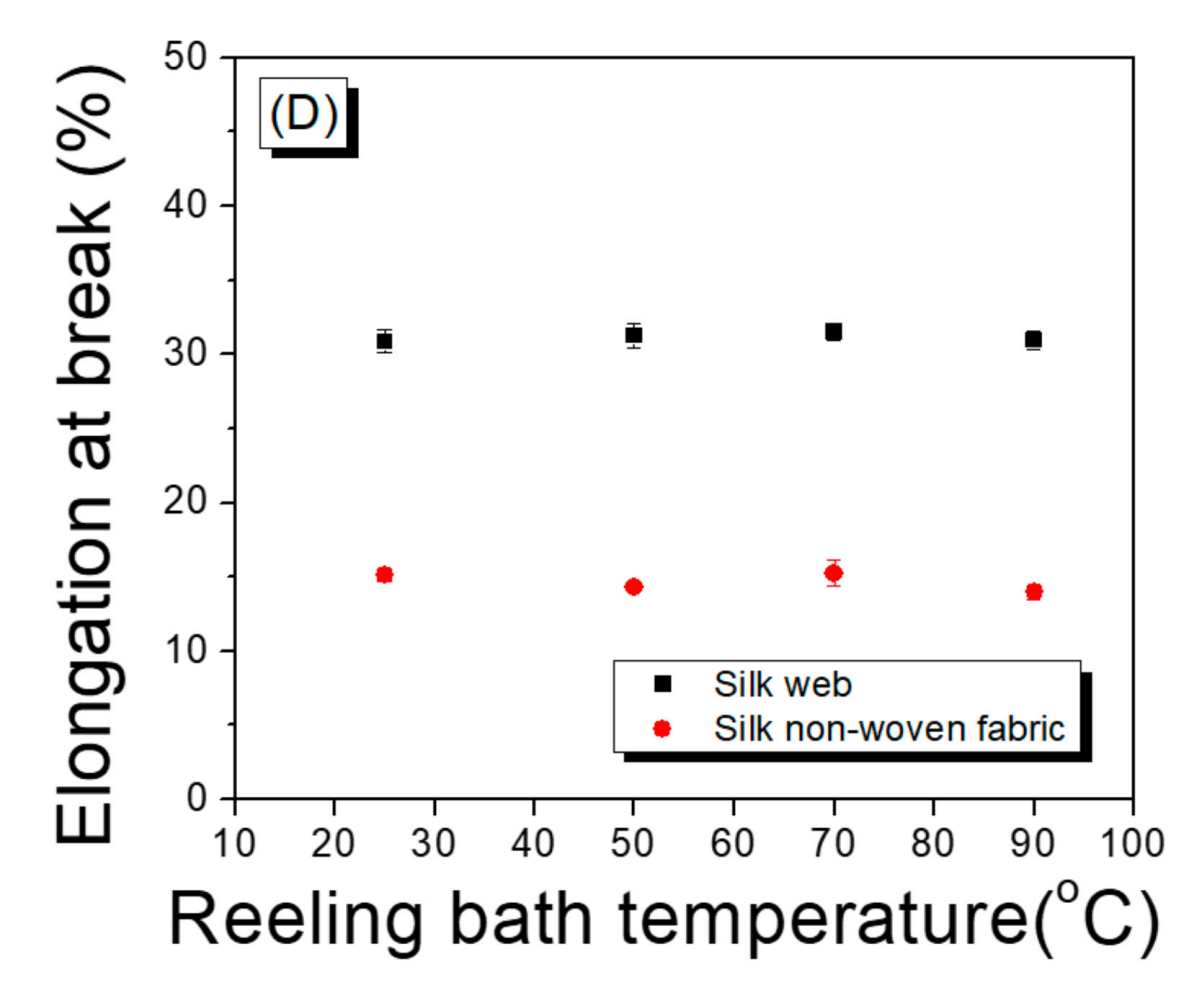
Representative stress–strain curves of the ((**A**) silk web and (**B**) silk non-woven fabric), (**C**) tensile strength, and (**D**) elongation at break of the silk web and silk non-woven fabrics prepared at various reeling bath temperatures. The reeling velocity was 39.2 m/min.

Figure 9 shows the effect of the reeling bath temperature and hot press treatment on the mechanical properties of the silk web and the silk non-woven fabric. The reeling bath temperature had almost no effect on the mechanical properties of the web and the non-woven fabric, and similar S–S curves were obtained. On the other hand, the hot press treatment had a strong influence on the S–S curves of silk porous materials. That is, the web exhibited the behavior of ductile material with high elongations at break (>30%), regardless of the reeling bath temperature. The inset in Figure 9A reveals the unclear fracture shape of the silk web, indicating that the breakage occurs consecutively. This might have been due to (1) the sericin bindings between the silk filaments and (2) the silk filaments themselves being gradually broken in the web by tensile force. This led to an increase in the high elongation at break. In the case of the silk non-woven fabrics, behavior consistent with a stiff material was observed. That is, abrupt breakage of the filaments occurred, owing to the strong sericin binding between the silk filaments, regardless of the reeling bath temperature. This is confirmed by the inset of Figure 9B, which reveals the relatively clear fracture shape of the non-woven fabric. In other words, the different behaviors between silk web and silk non-woven fabric with tensile force are attributed to the different binding strengths of sericin in silk web and non-woven fabric. That is, the highly bound silk filaments in non-woven fabric resisted all together against the external force (i.e., tensile force) and surrendered at a time resulting in an abrupt decrease of stress (Figure 9B). On the other hand, the less bound silk filaments in the silk web were broken one by one with the tensile force, showing a gradual decrease in stress (Figure 9A). 

In Figure 9C,D, the tensile strength (the highest stress) and elongation at break of the silk web and silk non-woven fabric are plotted as a function of the reeling temperature. The tensile strength and elongation of the fabrics were higher and lower, respectively, than those of the webs. When silk filaments were reeled from the silkworm cocoon and wound on the roll collector, the swollen sericin of the filament in the reeling bath helped to bind the filaments together. This allowed the formation of a silk web with desirable mechanical properties. When the web was hot pressed, the sericin was deformed, thereby strengthening the bonds between neighboring silk filaments [35,56]. The higher strength and lower elongation of the silk non-woven fabric (compared with those of the silk web) resulted mainly from the increased sericin binding effect induced by the hot press treatment. 

In addition, the tensile strengths and breaking elongations of silk web (30~40 MPa and 30~35%, respectively) and silk non-woven fabric (60~70 MPa and 10~15%, respectively) were much higher than those of electro-spun silk web (10~11 MPa and ~5%, respectively) [44]. This might be attributed to (1) that silk web and non-woven fabric consist of raw silk filament, (2) that sericin binds the silk filaments in the silk web and non-woven fabric, and (3) that silk filaments are arranged regularly in silk web and non-woven fabric. On the contrary, regenerated silk fibers (i.e., lower MW and less crystallized than raw silk [27,28,29,30,31]) were randomly arranged, without a binder, in the electro-spun silk web. 

Unlike the effect of the hot press treatment, the reeling bath temperature had negligible effects on the mechanical properties of the silk web and silk non-woven fabric. This resulted from the fact that the reeling bath temperature had almost no effect on the binding effects of the sericin between the silk filaments. That is, the reeling bath temperature affected the swelling of the sericin, which determined the detachment of the silk fibroin from the silkworm cocoon. However, when the sericin of silk filament was swollen enough to detach from the cocoon, it seemed that the swollen sericin at different temperatures almost did not affect the binding force between the silk filaments, resulting in the similar mechanical properties of the silk web. 

## 4. Conclusions

In the present study, the mechanical preparation of a natural silk web and a silk non-woven fabric, for their mass production, were successfully achieved using an electric winder system. To understand the effects of process variables on the preparation, structure, and properties of the web and non-woven fabric, these materials were prepared under different conditions: reeling velocity variation, hot press treatment, and reeling bath temperature variation. 

The reeling velocity had no effect on the crystallinity, appearance, and filament arrangement of the silk but exerted a strong influence on the mechanical properties, reel-ability, porosity, and swelling ratio of the silk web. From the viewpoint of mechanical properties and productivity, a reeling velocity of 39.2 m/min represented the optimal condition for silk web preparation. 

The hot press treatment led to considerable changes in the porosity and mechanical properties of the silk web. The reeling bath temperature has a significant effect on the reel-ability of the silk filament from the cocoon, and the lowest temperature for good reel-ability was 50 °C. The effect of the bath temperature on the structure and properties of the silk porous materials varied with the form (i.e., web or non-woven fabric) of the material. In the case of the silk web, as the reeling bath temperature was increased, the β-sheet crystallites were slightly more formed, whereas the porosity and swelling ratio decreased slightly. However, the structure and properties of the silk non-woven fabric remained unchanged with changes in the bath temperature. 

This study revealed that (1) an electric winder can be effectively used for the mechanical preparation of a silk web and a silk non-woven fabric, and (2) the structure and properties of porous silk materials can be diversely changed by controlling the reeling velocity, reeling bath temperature, and hot press treatment. Non-textile applications including biomedical and cosmetic applications require diverse performances tailored to the application of interest. The findings of the present study can be utilized as basic knowledge that contributes to the eventual commercialization of porous silk materials for various applications. Especially, it is expected that the silk web and silk non-woven fabric in this study can be utilized for developing various biomedical and cosmetic products. For this, it is thought that further studies should be conducted in the future to reduce production costs and to evaluate the performance of silk web and non-woven fabric as biomedical and cosmetic products. 

## Figures and Tables

**Table 1:** Photographs of 20 mm (width) × 50 mm (height) silk webs prepared at various reeling velocities. Reeling bath temperature was 50 °C.

Indication of Cross-Over Angle (30°)	Reeling Velocity (m/min)		
	17.5	39.2	52.2
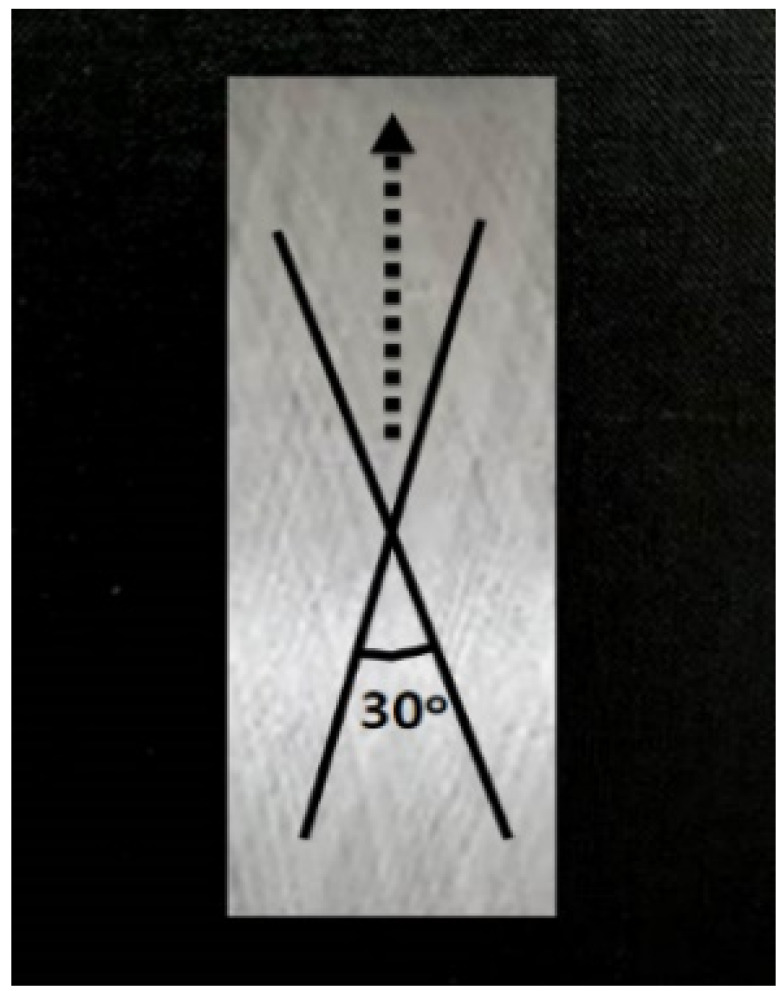	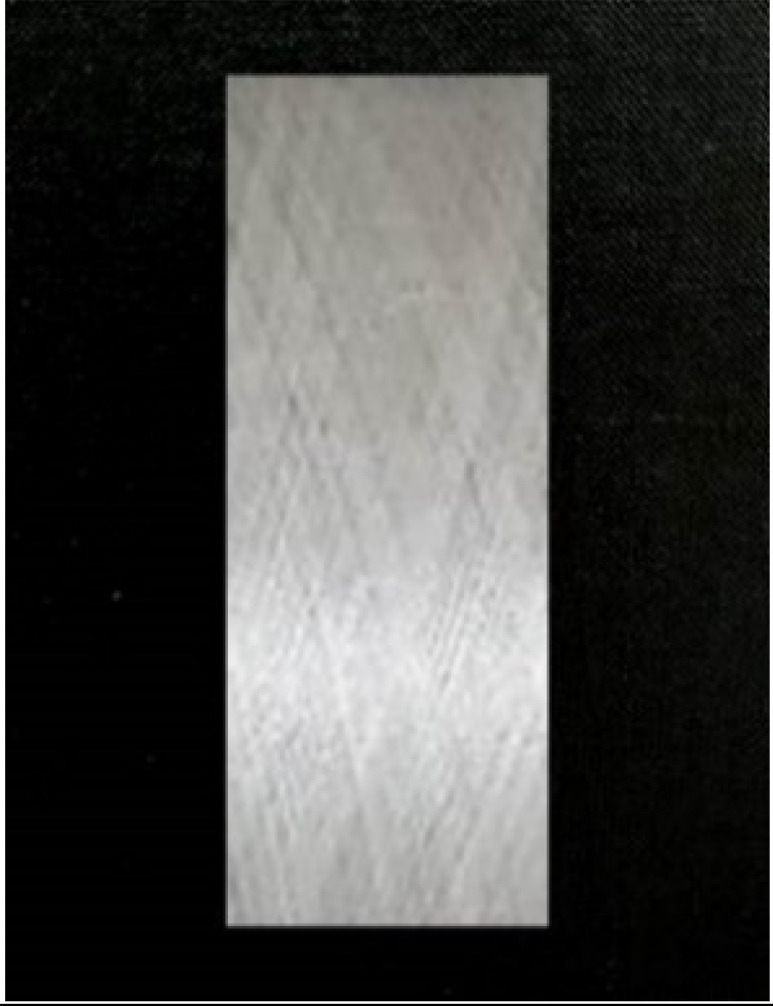	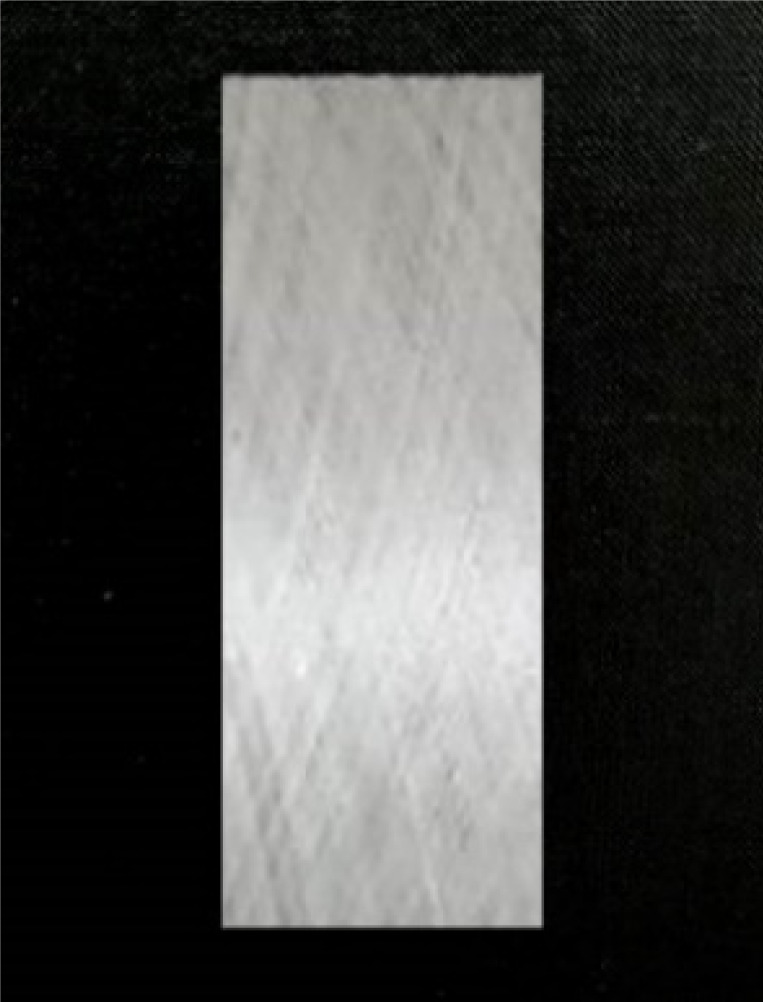	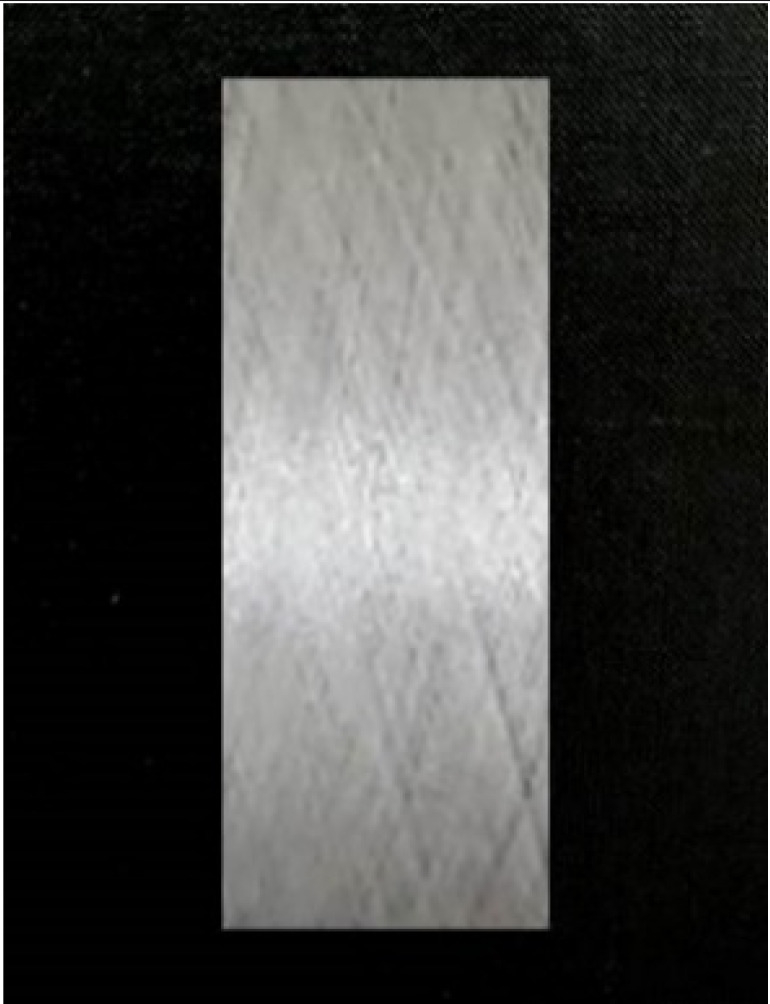

**Table 2:** FE-SEM images of the silk webs prepared at various reeling velocities. Reeling bath temperature was 50 °C.

Reeling Velocity (m/min)		
17.5	39.2	52.2
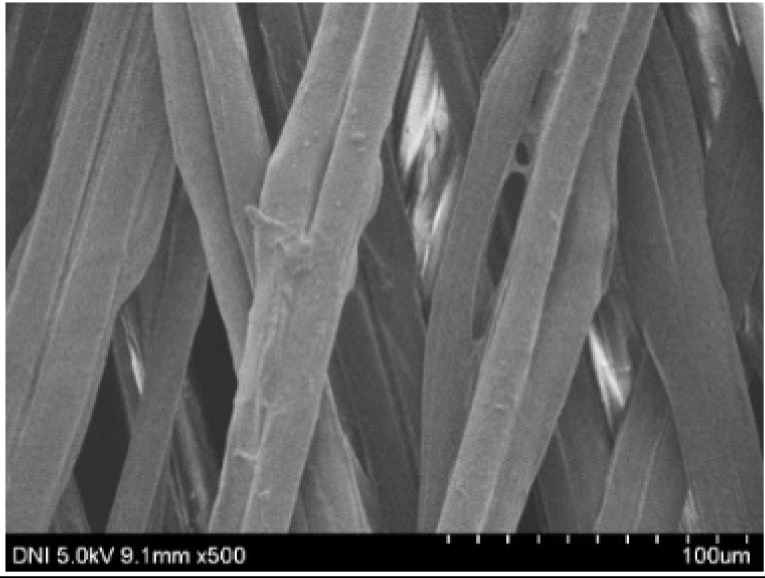	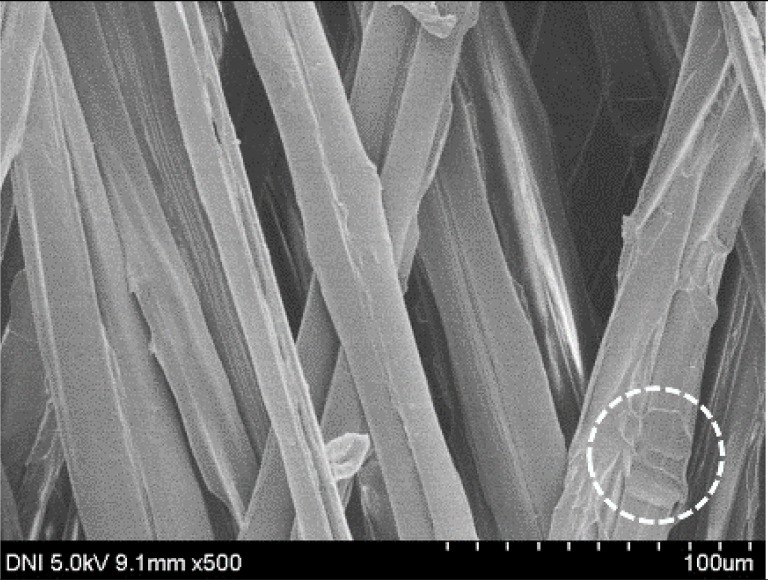	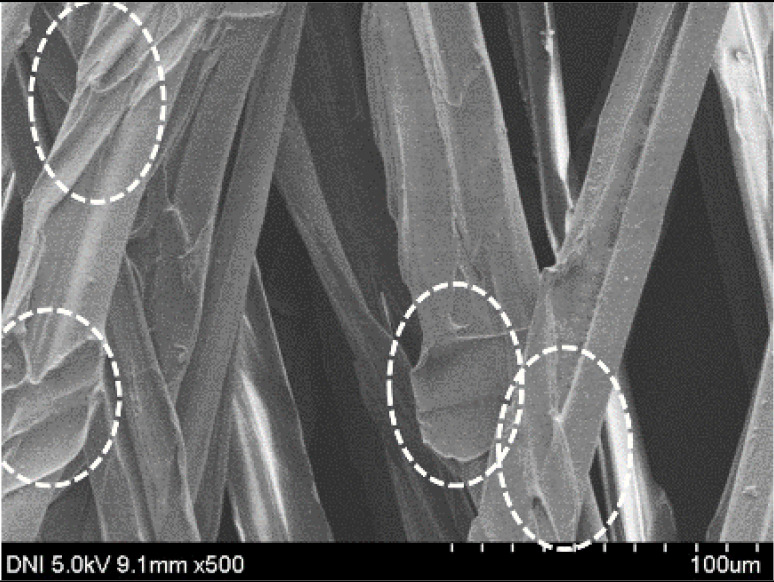

**Table 3:** Photographs of 20 mm (width) × 50 mm (height) silk web (untreated) and silk non-woven fabrics (hot-pressed silk web) prepared at various reeling bath temperatures. Reeling velocity was 39.2 m/min.

	Reeling Bath Temp. (°C)	25	50	70	90
Sample					
Silk web		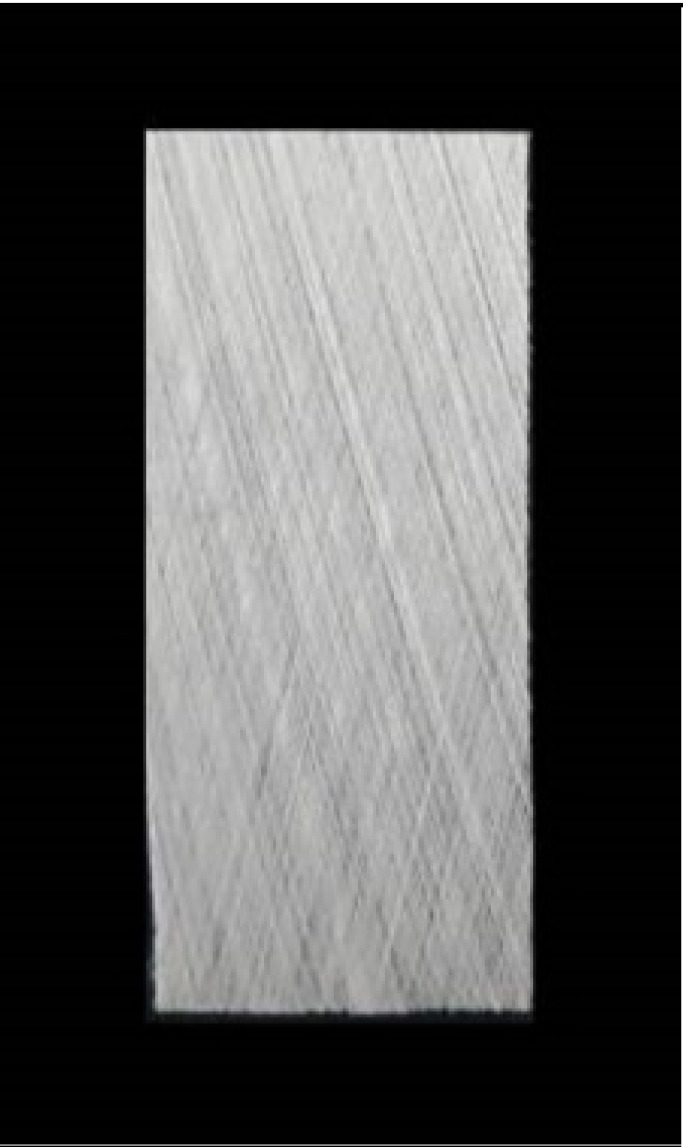	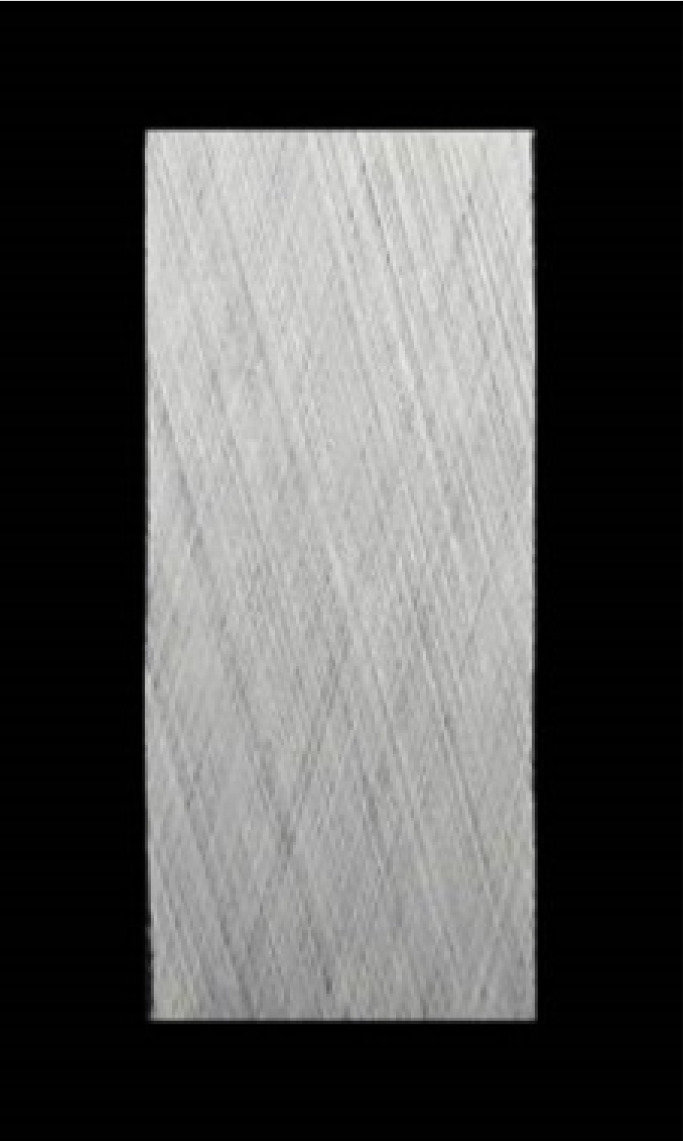	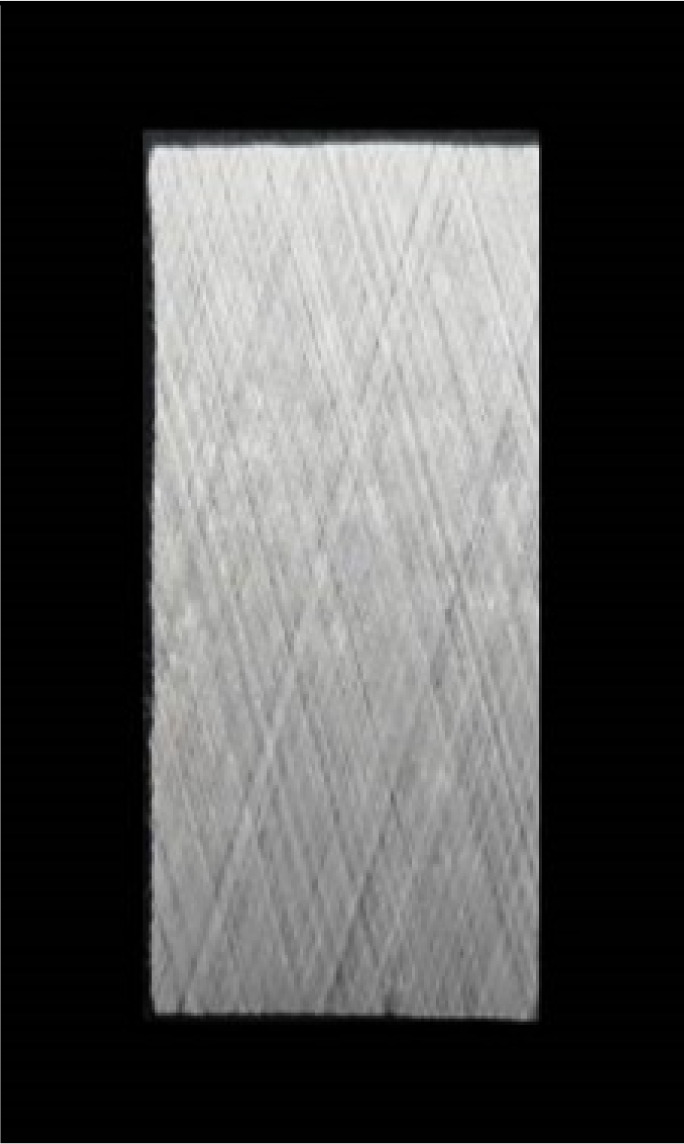	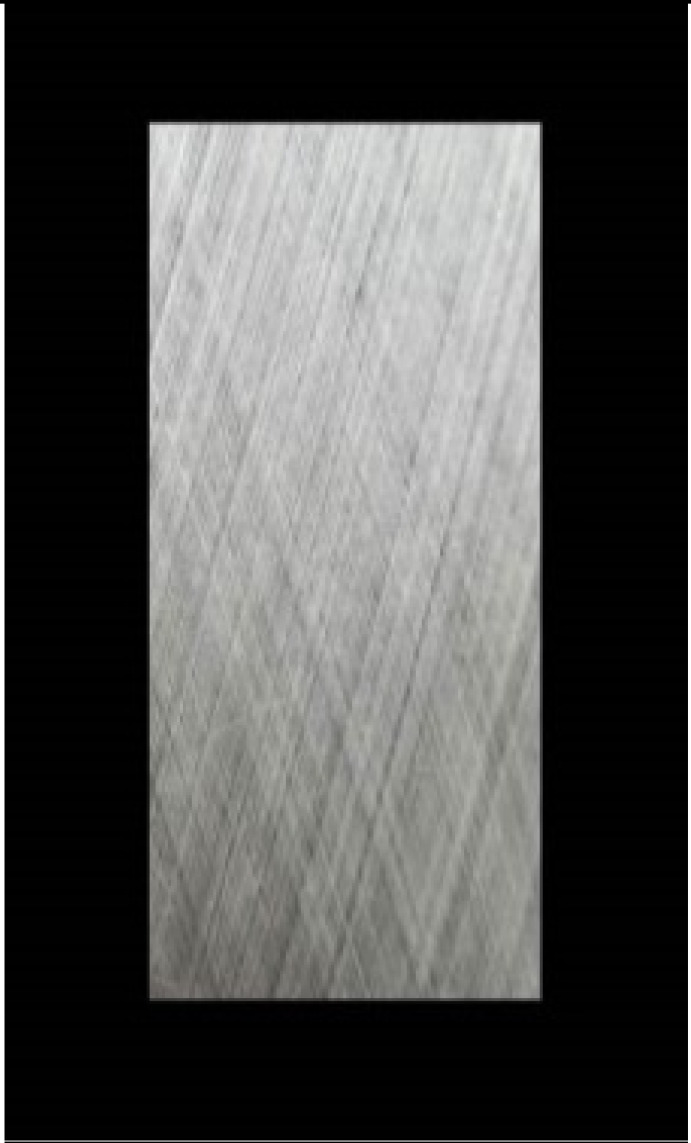
Silk non-woven fabric		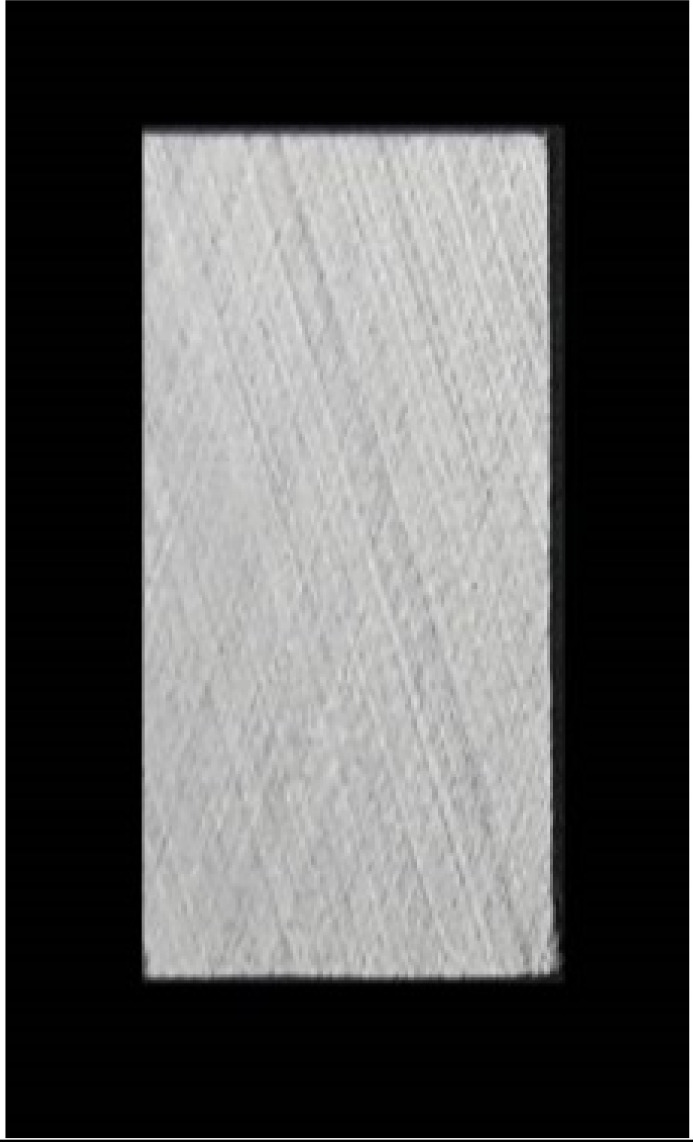	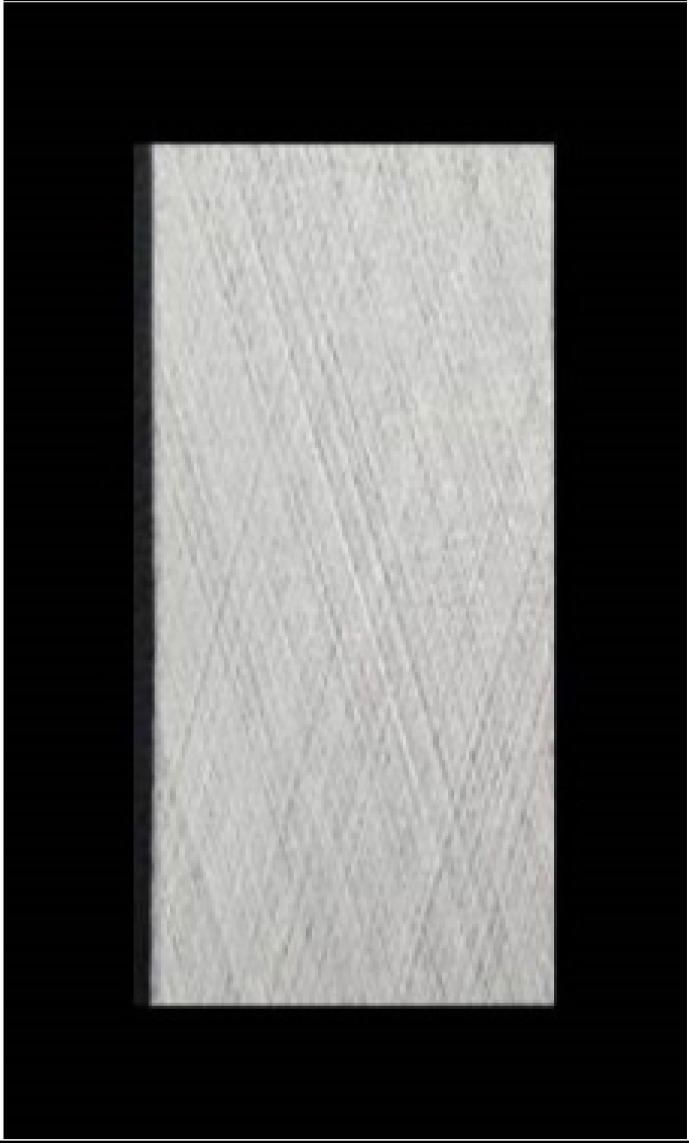	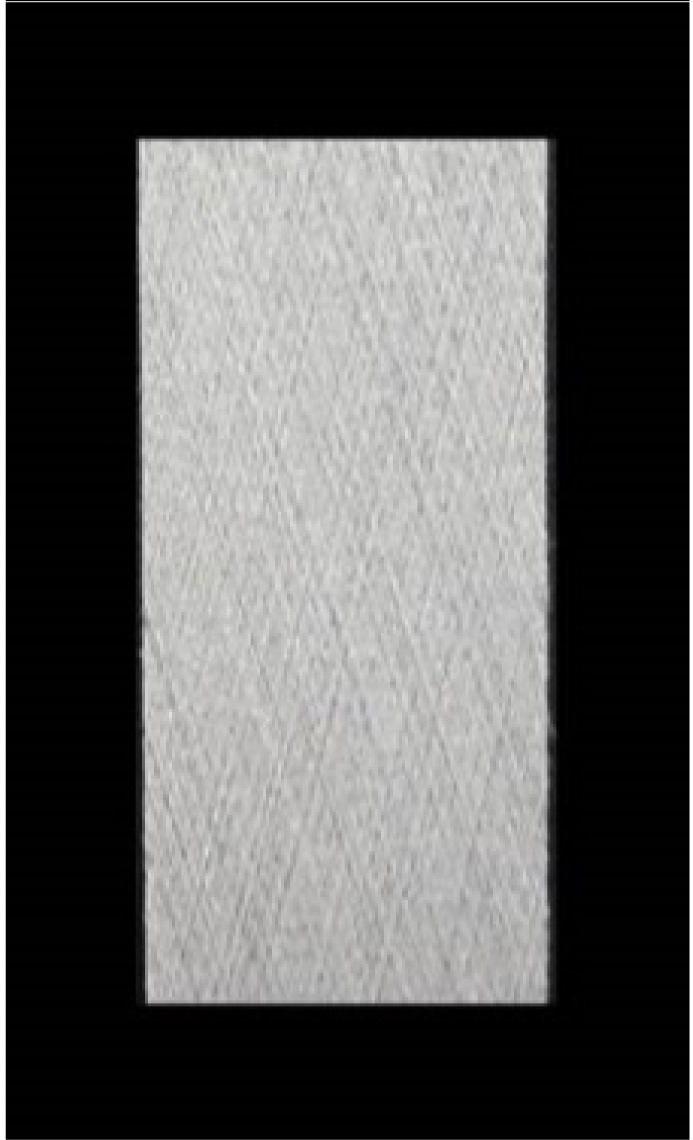	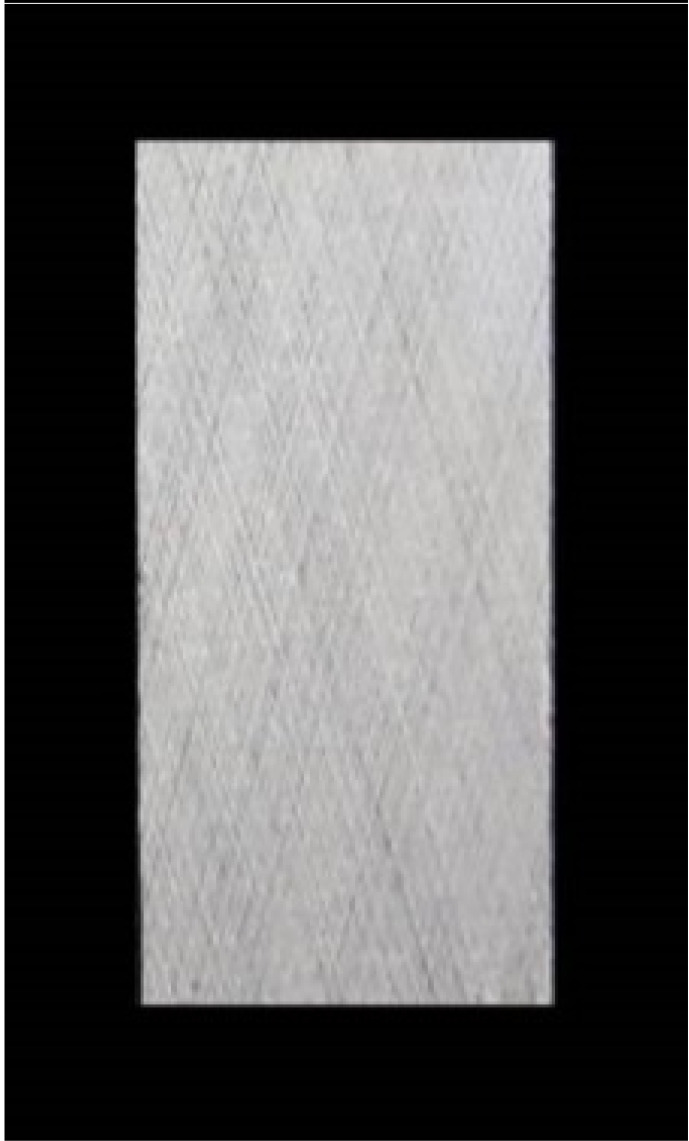

**Table 4:** FE-SEM images of silk web and silk non-woven fabrics prepared at various reeling bath temperatures. Reeling velocity was 39.2 m/min.

	Silk	Silk Web	Silk Non-Woven Fabric
Reeling Bath Temp. (°C)			
25		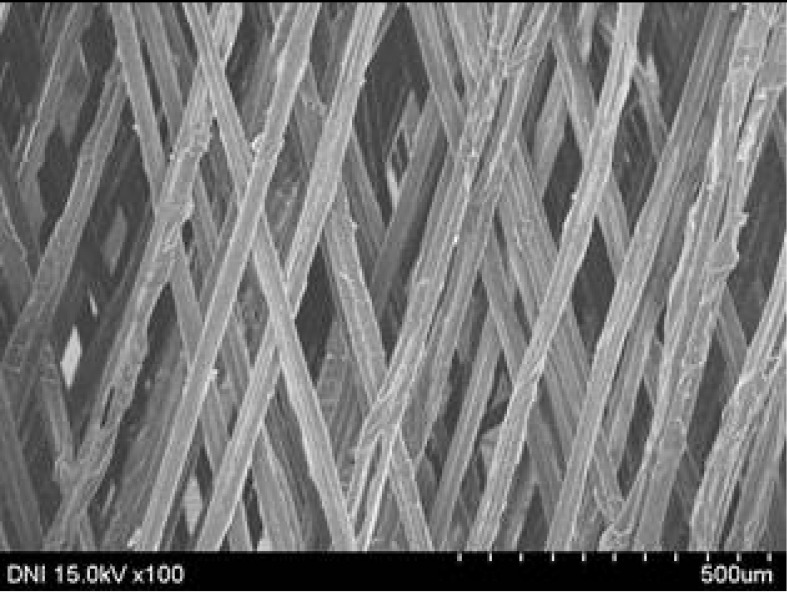	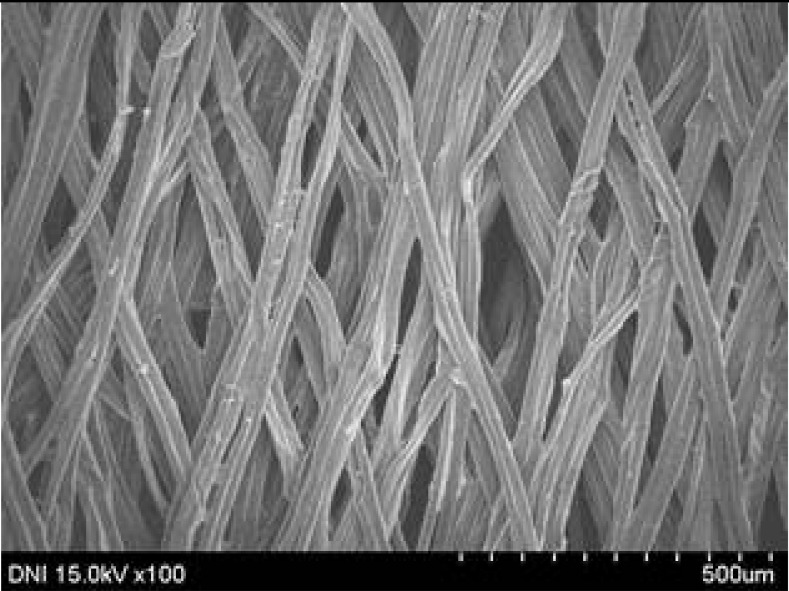
50		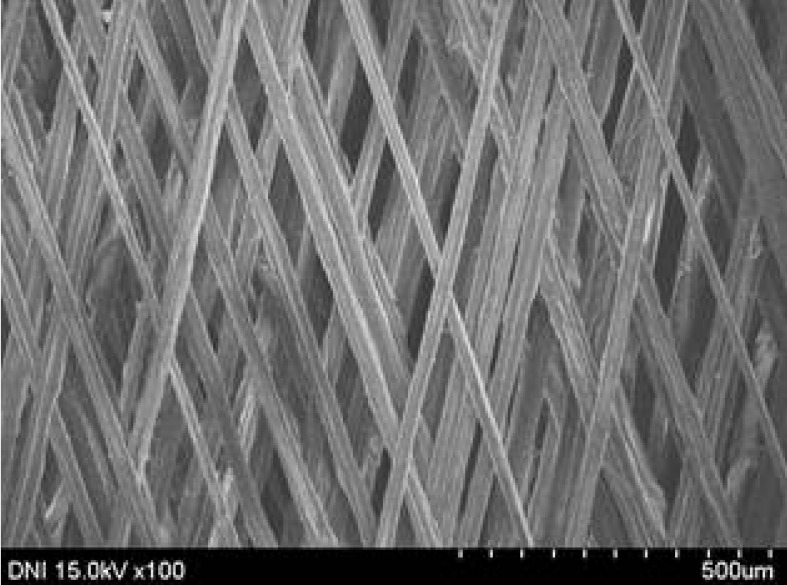	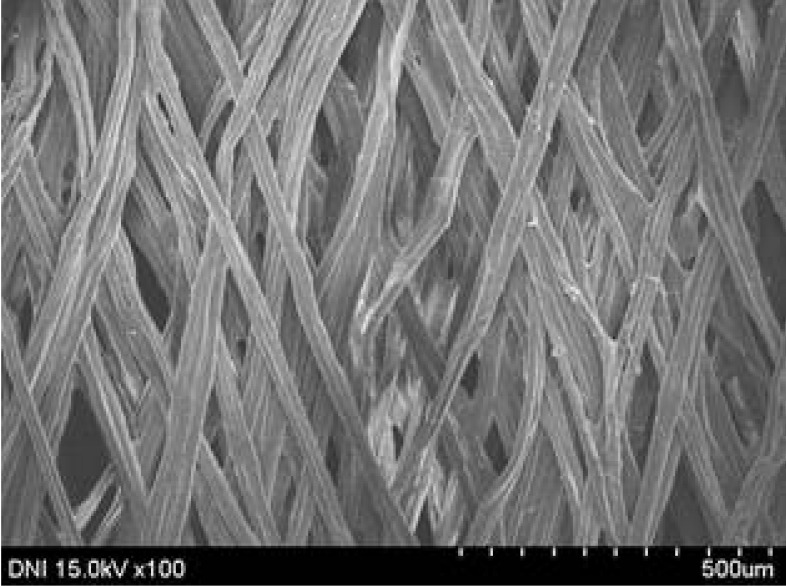
70		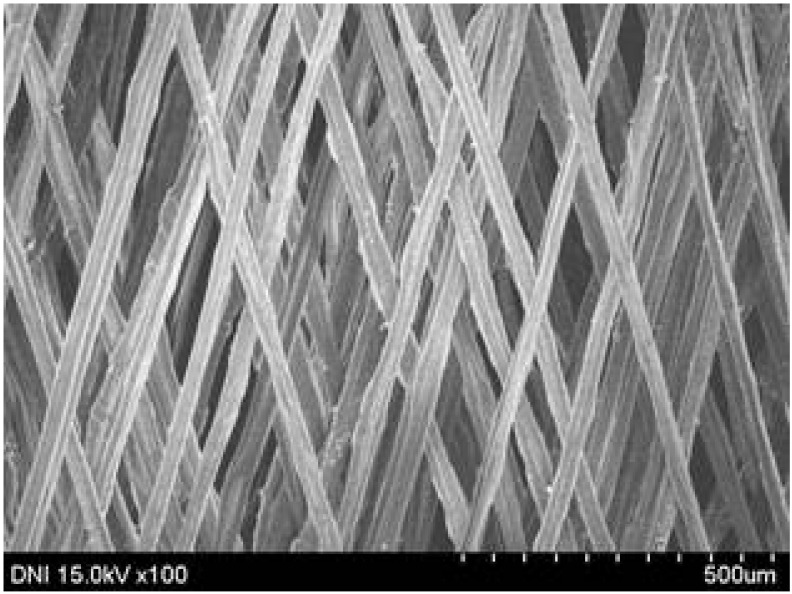	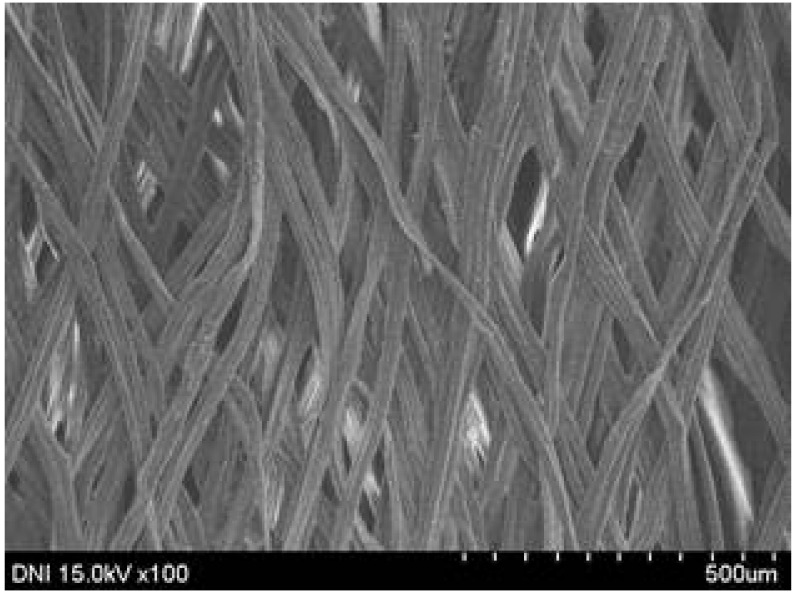
90		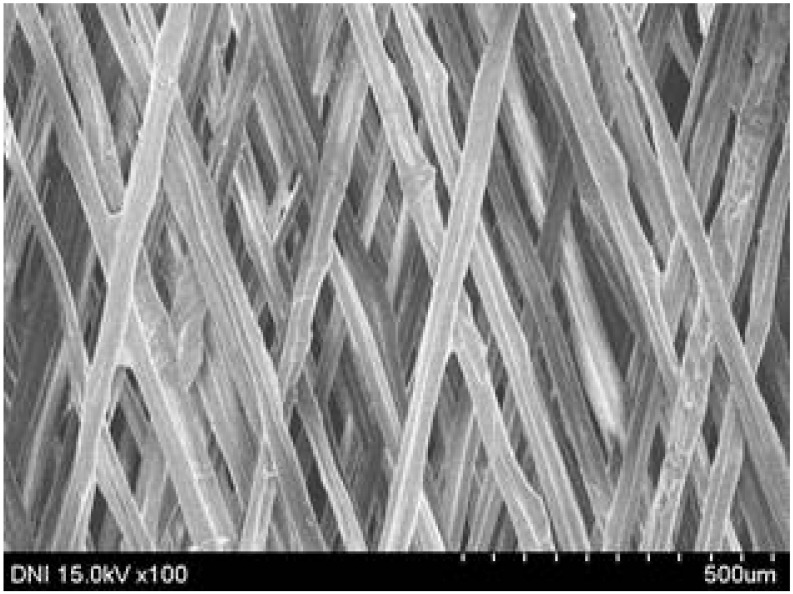	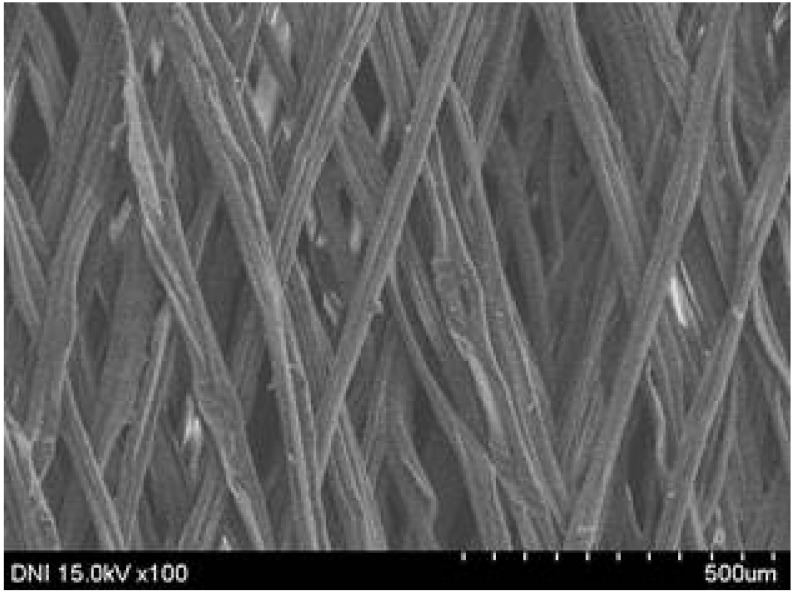

**Table 5:** X-ray fiber diffraction patterns of silk web and silk non-woven fabrics prepared at various reeling bath temperatures. Reeling velocity was 39.2 m/min.

	Silk Samples	Silk Web	Silk Non-Woven Fabric
Reeling Bath Temp. (°C)			
25		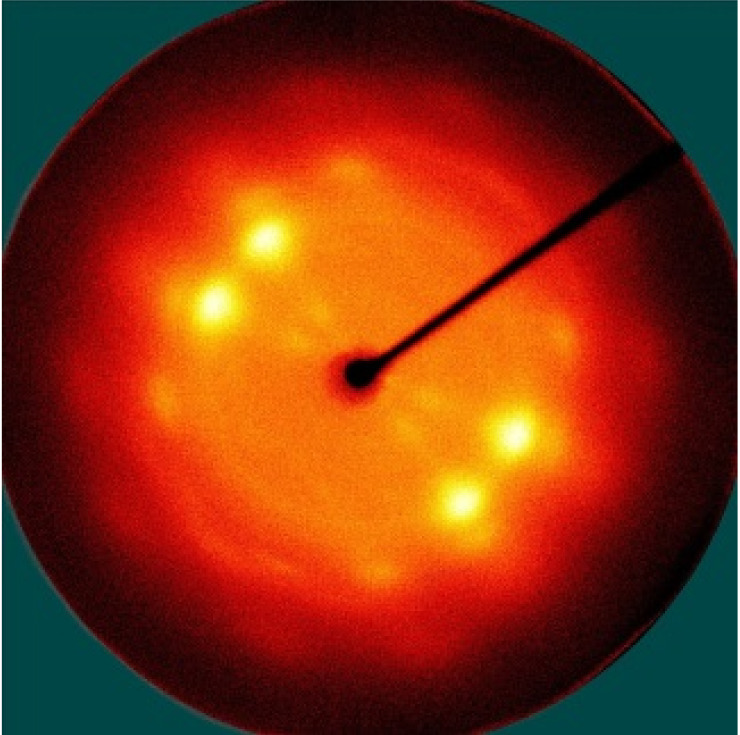	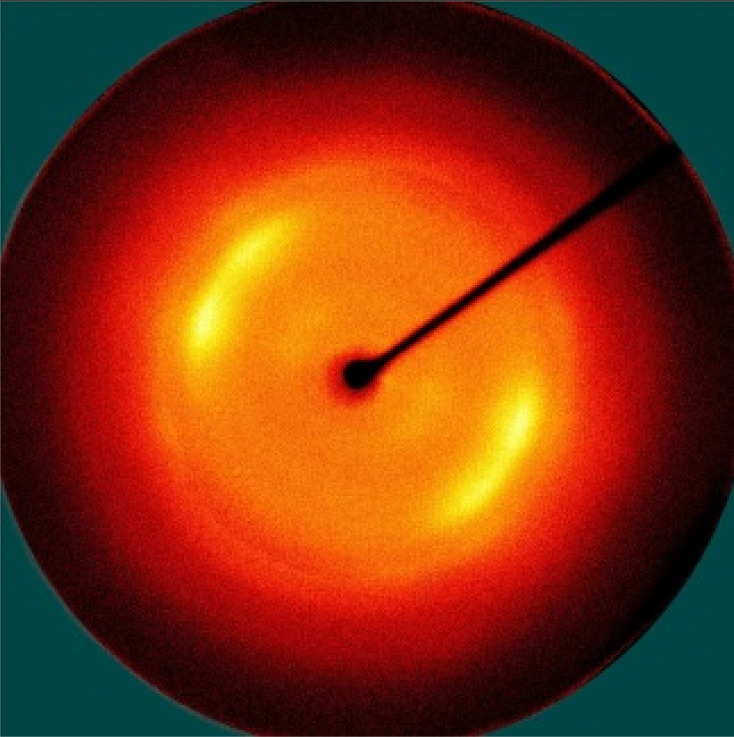
50		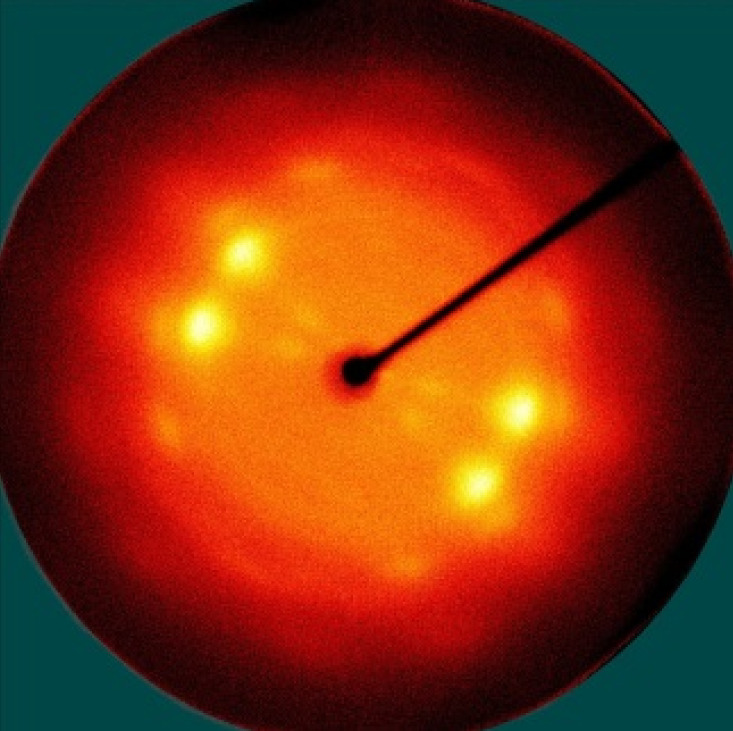	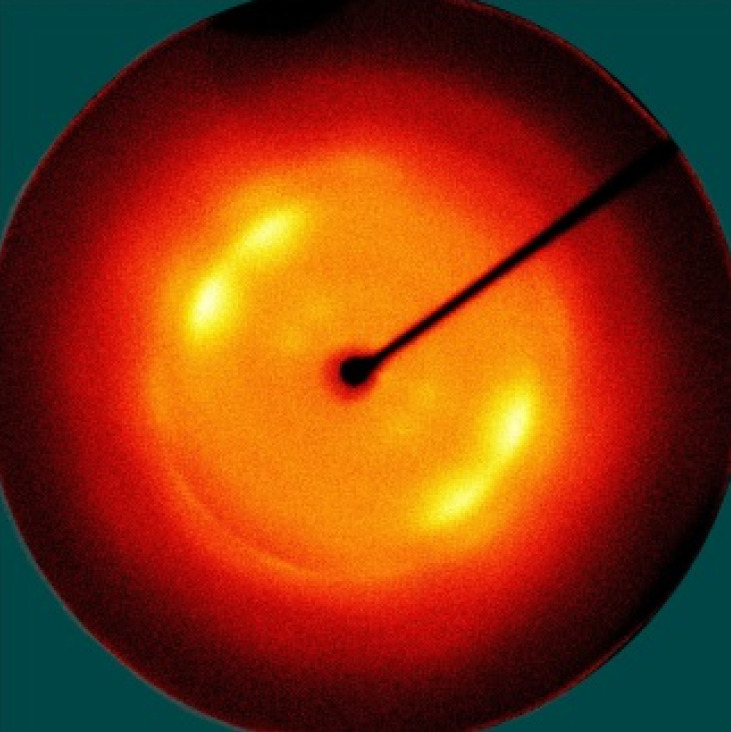
70		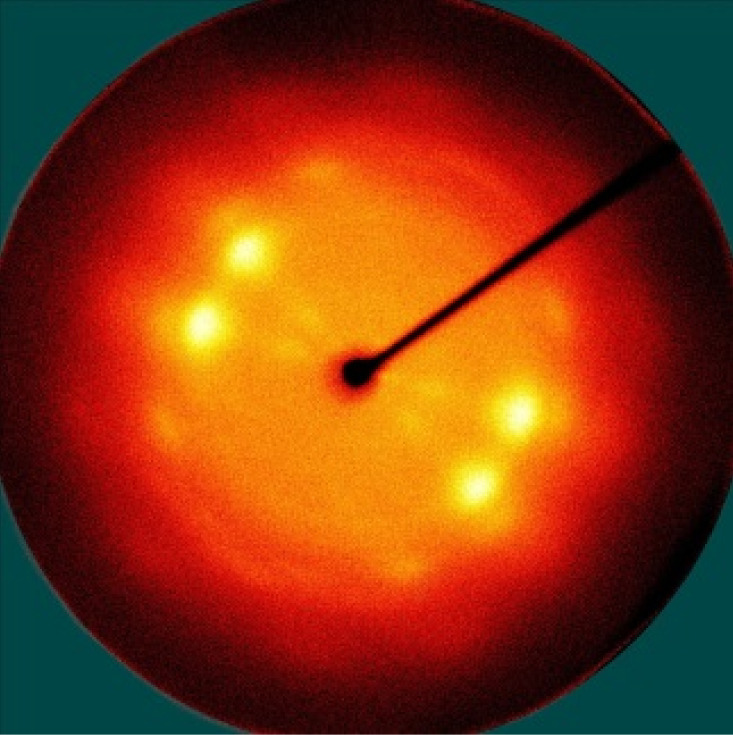	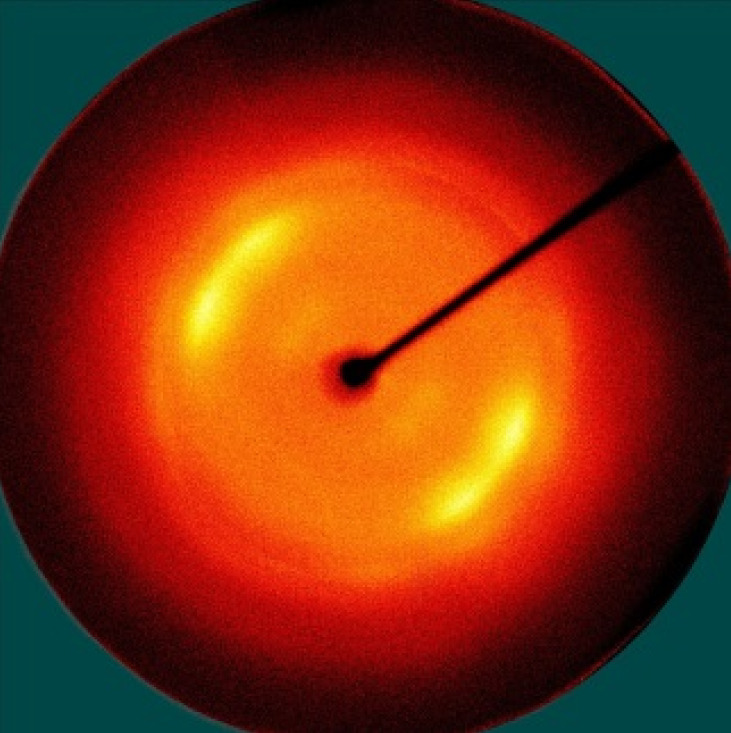
90		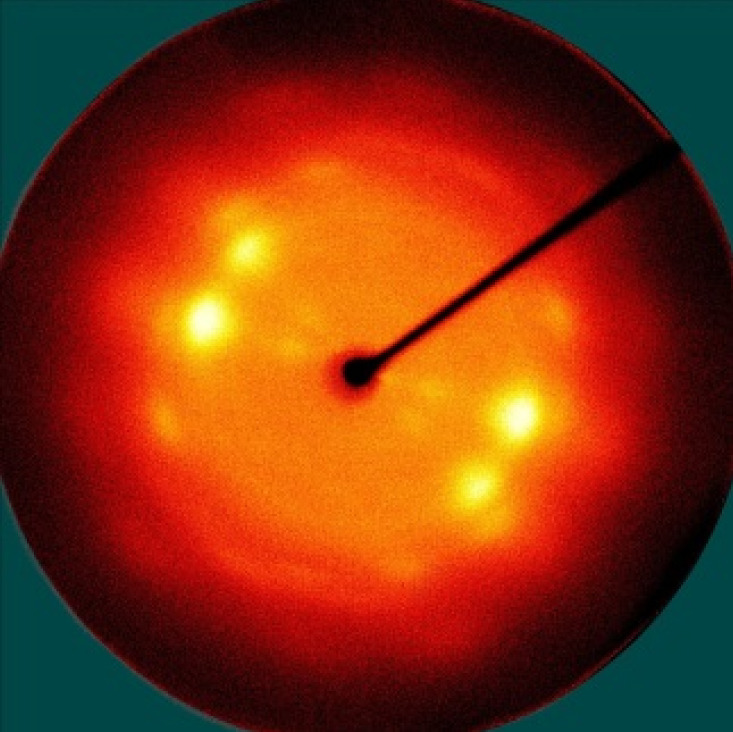	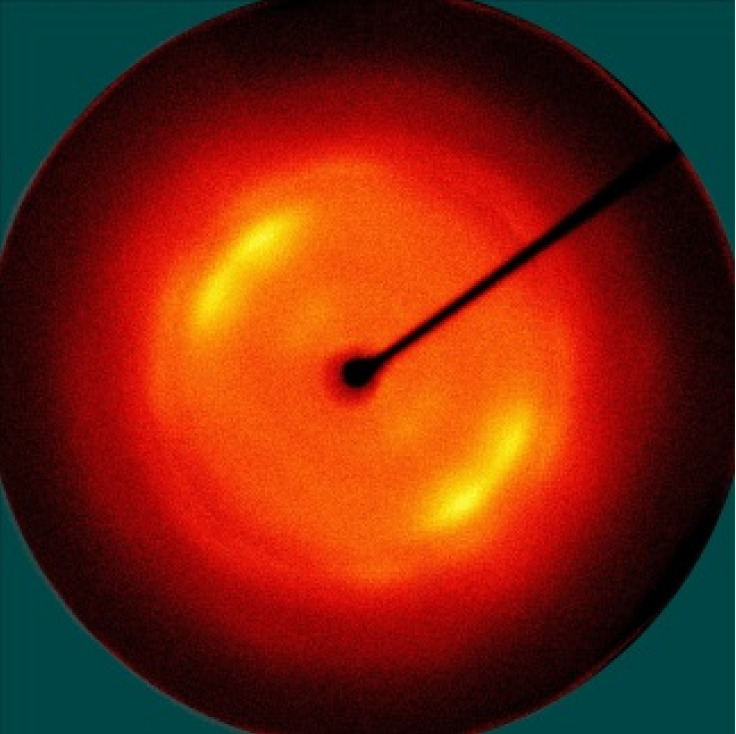

## Data Availability

The data presented in this study are available on request from the corresponding author.
